# Metrnl protects intestinal barrier function by regulating tight junctions via the IKKβ/IκBα/NFκB/MLCK/MLC signaling pathway

**DOI:** 10.1038/s41420-025-02457-1

**Published:** 2025-04-08

**Authors:** Zhi-Yong Li, Heng-Yu Luo, Fei Xu, Yao Xu, Chun-Hui Ma, Sai-Long Zhang, Sheng Xu, Yuan-Yuan Ma, Nan Li, Chao-Yu Miao

**Affiliations:** 1 Department of Pharmacology, Second Military Medical University/Naval Medical University, Shanghai, 200433 China; 2 Department of Pathology, Faculty of Medical Imaging, Second Military Medical University/Naval Medical University, Shanghai, 200433 China; 3 Department of Immunology, Second Military Medical University/Naval Medical University, Shanghai, 200433 China; 4 Senior Department of Hematology, The Fifth Medical Center of People’s Liberation Army(PLA), General Hospital, Beijing, 100010 China

**Keywords:** Intestinal diseases, Translational research

## Abstract

Meteorin-like (Metrnl), also known as Subfatin, IL-41, or Cometin, is a secreted protein predominantly expressed in the intestinal epithelium. The intestinal barrier, primarily consisting of epithelial cells connected by tight junctions, is essential for maintaining gut homeostasis by preventing harmful substances from entering the body. Despite Metrnl’s high expression in the intestine, its role in barrier function remains unclear. In this study, we investigated Metrnl’s role in intestinal barrier function using both loss-of-function (using global and intestinal epithelium-specific knockout mice) and gain-of-function (using intestinal epithelium-specific overexpression mice) approaches. Our findings showed that Metrnl deficiency disrupted tight junctions between enterocytes and exacerbated endotoxin-induced barrier dysfunction. Mechanistically, Metrnl deficiency triggered activation of the IKKβ/IκBα/NFκB signaling pathway, leading to increased MLCK expression and MLC phosphorylation. The NFκB inhibitor PDTC reversed this effect both in vivo and in vitro. Macrophages played an essential role in Metrnl’s intestinal barrier protective effects during endotoxemia, but were not necessary in burn-induced barrier injury, suggesting potential differences in mechanism between these conditions. Notably, recombinant Metrnl protein administration protected against barrier dysfunction, and genetic overexpression of Metrnl in enterocytes preserved barrier function and alleviated DSS-induced colitis. These findings establish Metrnl as a key regulator of intestinal barrier integrity through the IKKβ/IκBα/NFκB/MLCK/MLC pathway, highlighting its potential therapeutic value in treating barrier dysfunction disorders.

**Figure:**
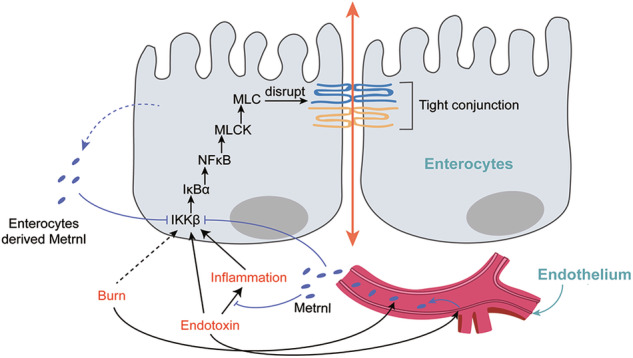
Intestinal barrier dysfunction triggers, such as endotoxin and severe burns, may induce the release of Metrnl from vascular endothelium. This leads to an increase in circulating Metrnl. Both circulating Metrnl and local Metrnl inhibit inflammation and the IKKβ/IκBα/NFκB/MLCK/MLC signaling pathway in enterocytes, thereby protecting tight junctions from disruption caused by endotoxin or burns.

Intestinal barrier dysfunction triggers, such as endotoxin and severe burns, may induce the release of Metrnl from vascular endothelium. This leads to an increase in circulating Metrnl. Both circulating Metrnl and local Metrnl inhibit inflammation and the IKKβ/IκBα/NFκB/MLCK/MLC signaling pathway in enterocytes, thereby protecting tight junctions from disruption caused by endotoxin or burns.

## Introduction

Metrnl is a secreted protein with demonstrated multifaceted benefits, including promoting remodeling of white adipose tissue, enhancing lipid metabolism, mitigating adipose inflammation, and ameliorating obesity-induced insulin resistance [1–3]. Studies have also shown that Metrnl facilitates neurite outgrowth [4], augment muscle repair post-injury [5], and provide cardioprotective effects against infarction and doxorubicin toxicity [6, 7]. Recently, our group has discovered that Metrnl exhibits heightened expression in the intestinal epithelium [8], prompting us to investigate its potential function in this tissue.

The intestinal barrier plays a crucial role in maintaining gut health by serving as a selective gateway between the intestinal lumen and the body’s internal environment. Its disruption can lead to various digestive and systemic disorders, including colitis, nonalcoholic fatty liver disease, and systemic inflammatory response syndrome [9, 10]. The compromised integrity of the intestinal barrier permits the translocation of intestinal microflora, antigen and toxic substances into the underlying tissues and the systemic circulation from gut lumen, thereby triggering local and systemic inflammation response and organs dysfunction [11, 12].

To ascertain the role of Metrnl in the intestine, we generated intestinal epithelium-specific knockout mice for Metrnl and observed that Metrnl depletion in the intestinal epithelium exacerbates dextran sulfate sodium (DSS) induced experimental colitis [8, 13]. Nevertheless, the contribution of Metrnl to other intestinal functions, particularly intestinal barrier function, remains unresolved.

The intestinal barrier is a complex and highly regulated system that maintains intestinal homeostasis through physical, biochemical, and immunological defense mechanisms [14]. However, its function can be impaired by inflammation, enterocyte shedding, and damaged tight junctions [8]. Inflammation is a common cause of intestinal barrier dysfunction. Endotoxins, inflammatory factors, and other stimuli can trigger intestinal inflammatory responses and damage the barrier function [15]. Increased apoptotic shedding of enterocytes, which may involve the programmed cell death of intestinal epithelial cells, is also associated with intestinal barrier dysfunction [16, 17]. Additionally, damaged tight junctions can increase intestinal permeability, allowing harmful substances to enter the systemic circulation [14].

Tight junctions are located at the most apical position of the epithelial junctional complex. They are formed by the interaction between transmembrane proteins, such as claudins and occludins, and cytoskeletal proteins [14, 18]. The regulation of tight junctions involves various cellular processes and signaling pathways. One of the key regulators of tight junctions is myosin light chain kinase (MLCK), which is a calcium- and calmodulin-dependent kinase. By phosphorylating myosin light chain (MLC), a component of the actomyosin contractile apparatus, MLCK induces cytoskeletal contraction. This leads to the opening of the paracellular pathway and increased intestinal permeability [18, 19]. Tight junction disruption is a common feature of various pathological conditions, including ischemia-reperfusion, severe burns, and inflammation [14, 18, 19].

Notably, the modulation of the MLCK-MLC cascade involves various upstream signaling pathways that include the NFκB signaling pathway and the Rho family of small GTPases [20]. These pathways can be activated by an array of stimuli, such as pro-inflammatory cytokines and reactive oxygen species, ultimately leading to the disruption of tight junctions [15, 21, 22]. Therefore, gaining insight into the intricate mechanisms that regulate tight junctions and the MLCK-MLC pathway is crucial for developing novel therapies to prevent or treat various pathological conditions associated with tight junction dysfunction.

The present study aimed to investigate the role of Metrnl in intestinal barrier function. Given the high expression of Metrnl in intestinal epithelium and its known protective effects in other tissues [5–7], we hypothesized that it might play an important role in maintaining barrier integrity. We utilized multiple experimental approaches, including both loss-of-function and gain-of-function studies, to examine how Metrnl affects intestinal barrier function and to explore the underlying molecular mechanisms.

## Results

### Global knockout of Metrnl exacerbates endotoxin-induced intestinal barrier impairment

**Fig. 1:**
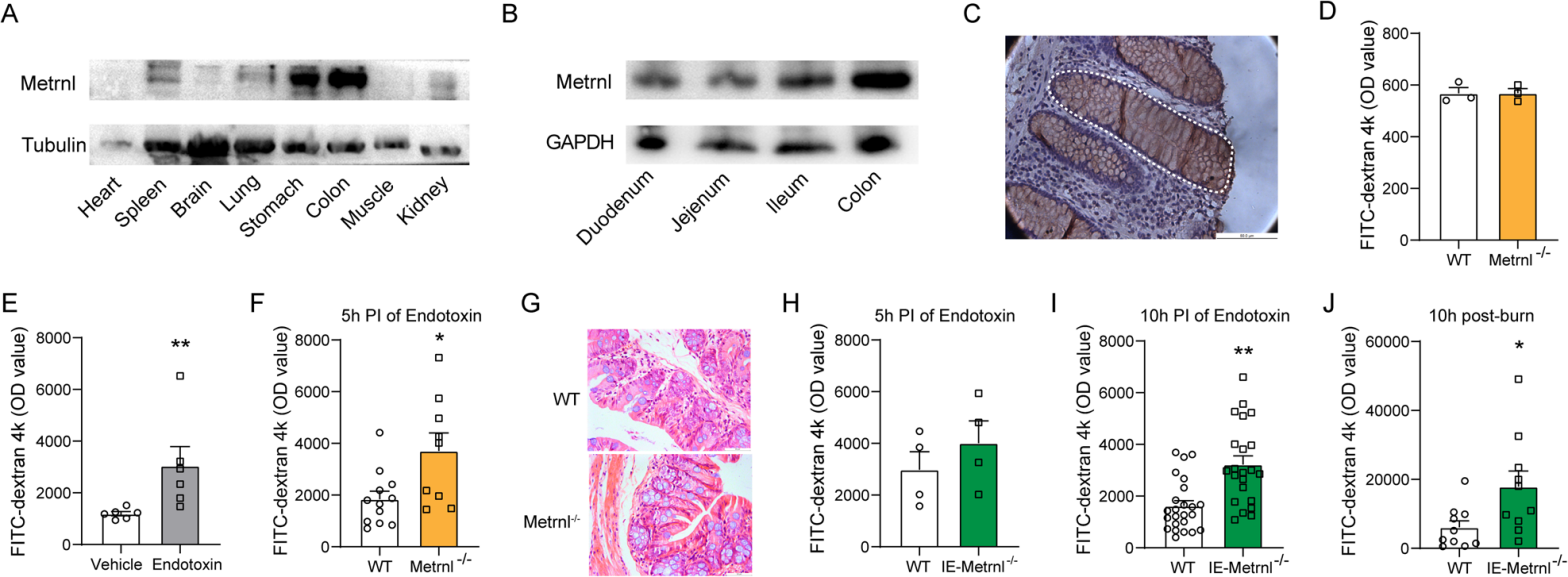
Metrnl expression in intestinal epithelial cells and its effects on intestinal barrier function. **A** Metrnl expression is most abundant in the gastrointestinal tract among the detected tissues. **B** Metrnl expression is detected in various parts of the intestinal tissue, with the highest expression found in the colon. **C** Metrnl is predominantly expressed in the colon epithelium. **D** Intestinal barrier permeability between global Metrnl knockout (Metrnl^−/−^) and wild type mice (WT) in normal condition. No significant differences were observed. *n* = 3. **E** Endotoxin-induced intestinal barrier dysfunction was evaluated at a dose of 5 mg/kg 5 hours post-injection (5 h PI) in C57 mice. *n* = 6. **F** Global knockout of Metrnl exacerbates endotoxin-induced intestinal barrier dysfunction. *n* = 12 for WT group and *n* = 9 for Metrnl^−/−^ group. **G** HE staining was performed on intestinal sections from Metrnl^−/−^ and wild type mice treated with endotoxin. No significant differences in intestinal morphology were observed between the two groups. **H** The effects of intestinal epithelial cell-specific Metrnl depletion (IE-Metrnl^−/−^) on intestinal barrier function 5 hours after endotoxin injection. *n* = 4. **I** Intestinal epithelial cell-specific knockout of Metrnl worsens endotoxin-induced intestinal barrier dysfunction 10 hours after endotoxin injection. *n* = 24 for WT group and *n* = 22 for IE-Metrnl^−/−^ group. The experiment was independently replicated twice. **J** Intestinal epithelial cell-specific knockout of Metrnl aggravates burn-induced intestinal barrier damage. *n* = 10. *P < 0.05, and **P < 0.01, compared with corresponding controls.

The expression of Metrnl protein was assessed in various tissues using Western blot analysis, and the results indicate that Metrnl is highly expressed in gastrointestinal tissue (Fig. 1A), consistent with prior findings on Metrnl mRNA levels [8]. Further, in the intestinal tissues spanning the duodenum, jejunum, ileum, and colon, it has been confirmed that Metrnl expression is most pronounced in the colon (Fig. 1B). Subsequent immunohistochemistry studies further revealed that Metrnl protein was predominantly located in the enterotypes (Fig. 1C).

We investigated the effect of Metrnl on intestinal barrier function using global Metrnl knockout mice (Metrnl^−/−^). FITC-dextran 4k was orally administered to evaluate intestinal permeability under normal conditions, showing no alterations (Fig. 1D). Endotoxin-induced intestinal barrier dysfunction is a common model for assessing intestinal barrier function in pathological states. We verified that intraperitoneal injection of endotoxin at a dose of 5 mg/kg induced intestinal barrier dysfunction 5 hours after injection (Fig. 1E). Intestinal permeability was measured in Metrnl^−/−^ mice treated with endotoxin. The fluorescence intensity of FITC in the blood of Metrnl^−/−^ mice was approximately two-fold higher than that in wild-type mice (Fig. 1F), indicating that global Metrnl deficiency exacerbates endotoxin-induced intestinal barrier impairment. Notably, no morphological differences were observed between Metrnl^−/−^ and wild-type mice in intestinal sections stained with HE (Fig. 1G).

### Intestinal epithelium-specific knockout of Metrnl exacerbates endotoxin-induced intestinal barrier impairment

To further investigate the contribution of intestinal epithelium in Metrnl deficiency-induced intestinal barrier impairment, IE-Metrnl^−/−^ mice were utilized to assess the impact of epithelial-specific Metrnl deletion on endotoxin-induced intestinal barrier function (Suppl. Fig. 1). Consistent with the protocol used in Metrnl^−/−^ mice, FITC fluorescence intensity was measured in the blood 5 hours after endotoxin administration, but no statistically significant difference was observed between IE-Metrnl^−/−^ and wild-type mice (Fig. 1H). However, at a later time point (10 hours after endotoxin administration), intestinal epithelium-specific Metrnl knockout mice exhibited a similar intestinal barrier impairment, with circulating FITC fluorescence intensity approximately two-fold higher than that in wild-type mice (Fig. 1I), thereby supporting the hypothesis that intestinal epithelium-mediated Metrnl deficiency exacerbates intestinal barrier impairment.

### Intestinal epithelium-specific knockout of Metrnl exacerbates burn-induced intestinal barrier impairment

In addition to endotoxemia, severe burn injuries can also result in intestinal epithelial barrier dysfunction [22]. To investigate the potential exacerbating effect of Metrnl deficiency on burn-induced intestinal barrier dysfunction, mice were subjected to burns under anesthesia and intestinal permeability was evaluated 10 hours later. The results showed that IE-Metrnl^−/−^ mice had significantly higher intestinal permeability than wild-type mice (Fig. 1J). This suggests that the impairment of the intestinal barrier related to Metrnl deficiency is caused not only by exogenous endotoxins but also by the stress associated with burn injury.

### Intestinal epithelium-specific deficiency of Metrnl results in changes in endotoxin-induced inflammation during intestinal barrier impairment

**Fig. 2:**
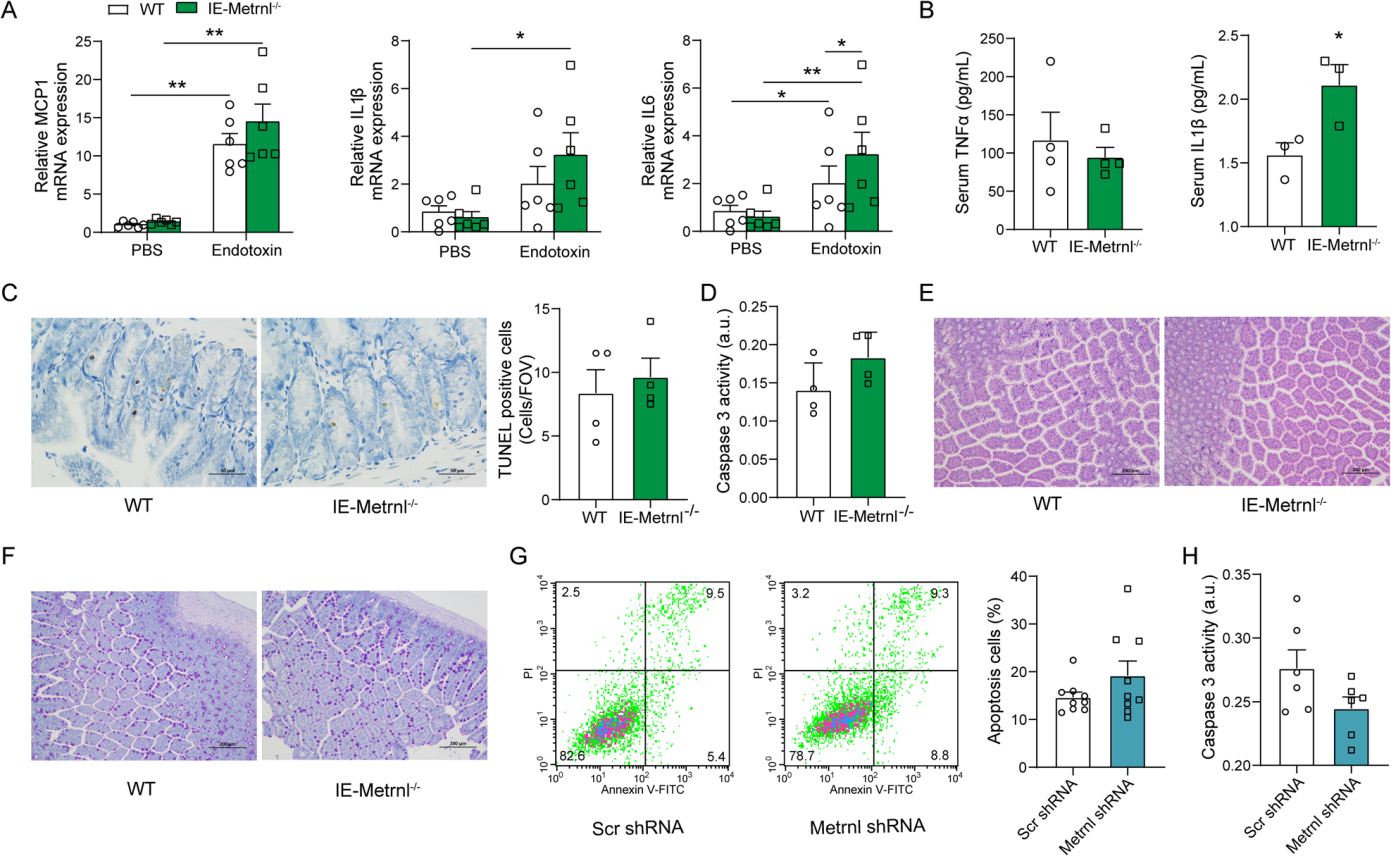
Metrnl deficiency amplifies the inflammatory response but does not affect intestinal epithelial apoptosis under endotoxin stimulation. **A** The intestine-specific knockout of Metrnl results in increased expression of IL6 in intestinal tissue when stimulated with endotoxin. *n* = 6. **B** The intestinal-specific knockout of Metrnl elevates the concentration of IL1β in the blood during endotoxin stimulation. *n* = 3-4. **C** Deletion of Metrnl in intestinal epithelial cells does not increase the number of apoptotic cells as observed by TUNEL staining. *n* = 4. The experiment was conducted twice. **D** Deletion of Metrnl in intestinal epithelial cells does not increase caspase 3 activity. *n* = 4. The experiment was conducted twice. **E**, **F** Comparison of intestinal morphology in IE-Metrnl^−/−^ and wild type mice treated with endotoxin. HE (**E**) and PAS (**F**) staining were performed on intestinal sections from IE-Metrnl^−/−^ and wild type mice 10 hours after endotoxin treatment. No significant differences were observed. **G** Knockdown of Metrnl in Caco2 cells does not increase the detection of annexin V-positive apoptotic cells. *n* = 9. **H** Knockdown of Metrnl does not increase caspase 3 activity in Caco2 cells. *n* = 6. The experiment was independently replicated twice. *P < 0.05, and **P < 0.01, compared with corresponding controls.

The Metrnl protein is involved in the inflammatory response and appears to have anti-inflammatory effects during inflammation [1, 23, 24]. To investigate whether inflammation is related to Metrnl deficiency-induced intestinal barrier impairment, we assessed the changes in inflammatory factors after intestinal epithelium-specific knockout of Metrnl treated with endotoxin. The findings show that although the knockout of Metrnl did not augment the expression of MCP1 and IL1β, it significantly increased the expression of IL6 in intestine (Fig. 2A). Upon examining blood inflammatory factors, we found that the TNFα concentration was unchanged, but the blood concentration of IL1β had increased (Fig. 2B). These suggest that the knockout of Metrnl amplifies both local and systemic inflammatory responses.

### Intestinal epithelium-specific Metrnl deficiency did not increase endotoxin induced intestinal epithelial cell apoptosis

To investigate the potential role of enterocyte apoptosis in Metrnl deficiency-induced intestinal barrier impairment, IE-Metrnl^−/−^ mice were treated with endotoxin, and enterocyte apoptosis was assessed by evaluating the number of TUNEL-positive cells and caspase 3 activation. No statistically significant differences were observed between IE-Metrnl^−/−^ and wild-type mice in either of these apoptosis indicators (Fig. 2C, D). Additionally, there was no evidence of enterocyte apoptotic shedding or mucous secretion alterations in the intestinal epithelium, as detected by HE and PAS staining (Fig. 2E, F). The impact of Metrnl on apoptosis was further investigated in Caco2 cells by silencing Metrnl using lentiviral vectors expressing small interfering RNA (Suppl. Fig. 2). Silencing Metrnl did not increase Annexin V-FITC positive Caco2 cells (Fig. 2G) or caspase 3 activity (Fig. 2H) when stimulated with endotoxin. These findings support the notion that Metrnl deficiency-induced intestinal barrier impairment is not caused by enterocyte apoptotic shedding.

### Metrnl deficiency exacerbates endotoxin-induced tight junction disruption between enterotypes

**Fig. 3:**
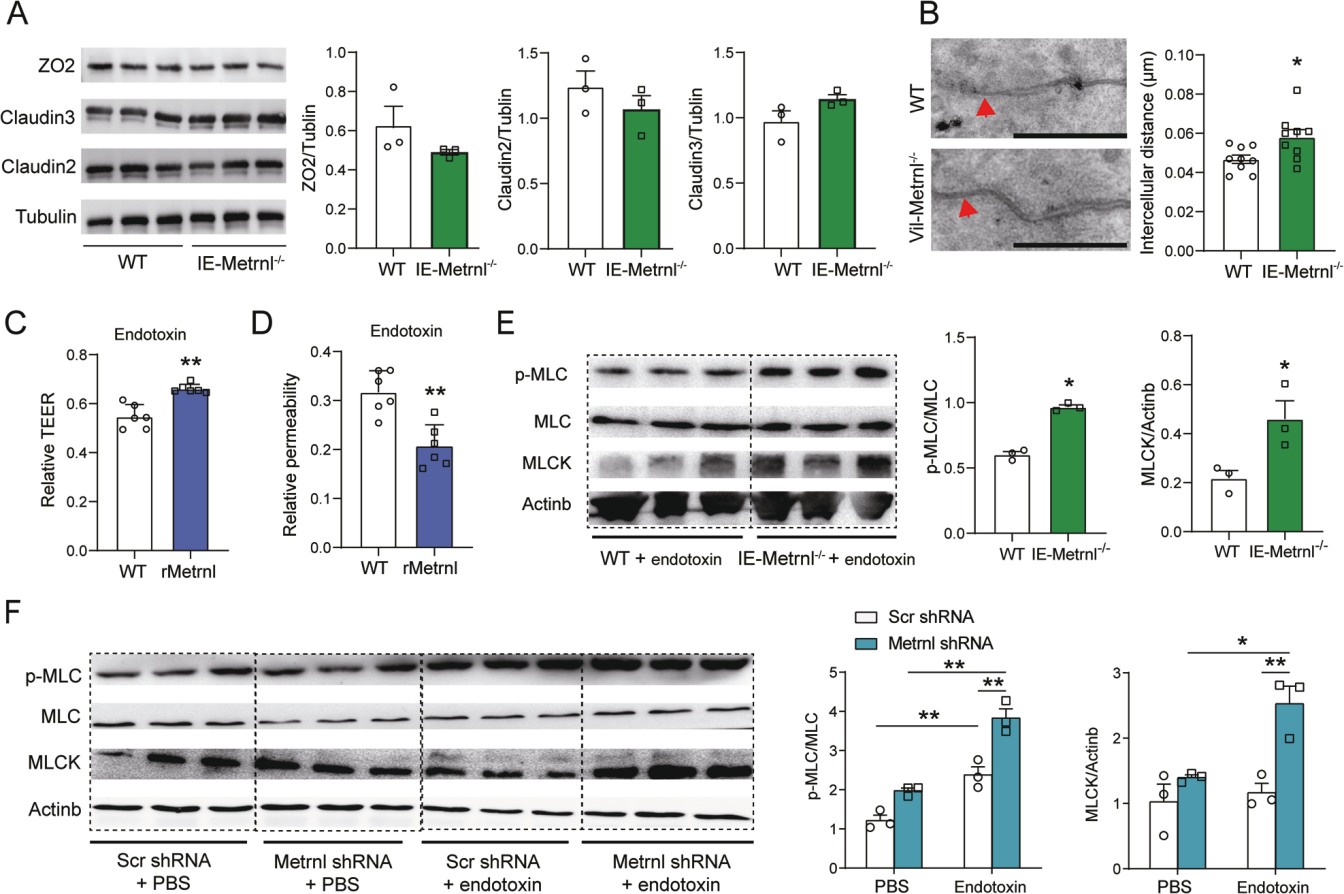
Impact of Metrnl deficiency on intestinal epithelial cells tight junction. **A** Immunoblot analysis of ZO2, Claudin2 and Claudin3 expression in intestine of IE-Metrnl^−/−^ and wild type mice. *n* = 3. **B** Representative transmission electron microscope images of IE-Metrnl^−/−^ and wild type mice. Bar = 1 nm. Arrow indicates tight junction between enterocytes. **C** Transepithelial electrical resistance (TEER) of Caco2 cells treated with or without Metrnl recombinant protein under endotoxin stimulation. *n* = 6. **D** Permeability of Caco2 cells to FITC-dextran 4k was measured with and without Metrnl recombinant protein under endotoxin stimulation. Relative permeability is defined as the ratio of the concentration of FITC-dextran in the upper chamber to the concentration of FITC-dextran in the bottom chamber. *n* = 6. The experiment was independently replicated twice. **E** Detection of myosin light chain kinase (MLCK) expression and phosphorylation of myosin light chain (MLC) with western blot of Caco2 cells treated with Metrnl shRNA and Scramble shRNA (Scr shRNA) mediated by lentivirus under endotoxin stimulation. *n* = 3. **F** Metrnl knockdown affects MLCK/MLC signaling in Caco2 cells treated with or without endotoxin. *n* = 3. *P < 0.05, and **P < 0.01, compared with corresponding controls.

To investigate the potential role of tight junctions in the intestinal barrier impairment induced by Metrnl deficiency, the expression levels of tight junction proteins ZO2, Claudin2 and Claudin3 were compared between IE-Metrnl^−/−^ and wild-type mice under endotoxin stimulation. However, no significant differences were observed (Fig. 3A). Similarly, the expression of these tight junction proteins was not affected by the absence of Metrnl in Caco2 cells under endotoxin treatment (Suppl. Fig. 3). Ultrastructural changes in thin sections of enterocytes were then evaluated using transmission electron microscopy. The results revealed that the disintegration of tight junctions between enterocytes was more pronounced in IE-Metrnl^−/−^ mice (Fig. 3B), indicating that the damage to tight junctions may contribute to the Metrnl deficiency-induced increase in intestinal permeability.

To further confirm the role of tight junctions in the enhancement of intestinal permeability induced by Metrnl deficiency, Caco2 cells were cultured in transwells to induce cell differentiation and to evaluate transmembrane potential with or without Metrnl recombinant protein. Metrnl protein significantly increased the transmembrane potential (Fig. 3C) and FITC-Dextran 4 K permeability under endotoxin treatment (Fig. 3D). Furthermore, the upregulation of MLCK expression and MLC phosphorylation were observed in Metrnl-deficient intestine (Fig. 3E) and in Metrnl silencing Caco2 cells treated with endotoxin (Fig. 3F). These findings suggest that the enhancement of intestinal permeability induced by Metrnl deficiency may be mediated through tight junctions, which are regulated by the MLCK/MLC signaling pathway.

### Overexpression of Metrnl specifically in the intestinal epithelium can alleviate endotoxin-induced intestinal barrier impairment

**Fig. 4:**
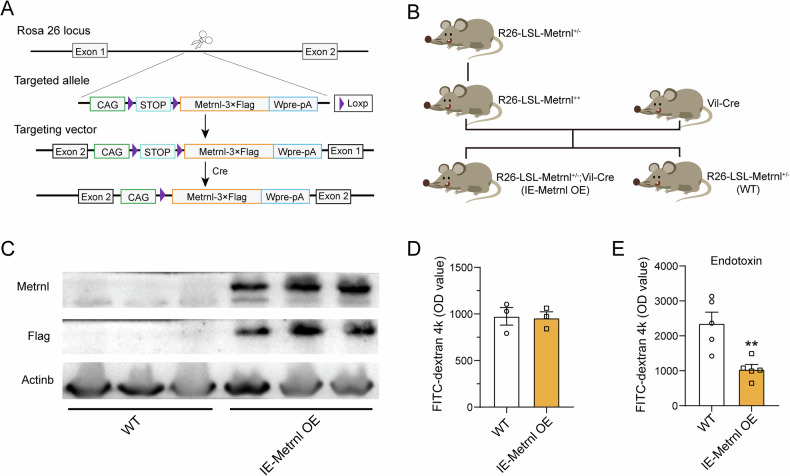
Enterocytes-specific overexpression of Metrnl improves endotoxin-induced intestinal barrier dysfunction. **A** Schematic diagram illustrating the method used to overexpress Metrnl. **B** The breeding strage for generating mice with intestinal-specific overexpression of Metrnl. **C** Verification of intestinal-specific overexpression of Metrnl in mice. **D** Overexpression of Metrnl in the intestine did not alter intestinal barrier function under normal conditions. *n* = 3. **E** Intestinal-specific overexpression of Metrnl improved endotoxin-induced intestinal barrier dysfunction. *n* = 5. **P < 0.01 compared with wild type (WT).

To investigate the potential therapeutic role of the Metrnl gene in endotoxin-induced intestinal barrier impairment, Metrnl-intestinal epithelium-specific transgenic mice (R26-LSL-Metrnl;Vil-Cre, IE-Metrnl OE) were generated by crossing Metrnl conditional overexpression homozygous mice with Vil-Cre mice (Fig. 4A, B). Metrnl overexpression in the intestine was confirmed via Western blot using anti-Metrnl and anti-Flag antibodies (Fig. 4C). Under normal conditions, no significant differences in intestinal barrier function were observed between IE-Metrnl OE and wild-type mice (Fig. 4D). However, after endotoxin treatment, intestinal-specific overexpression of Metrnl significantly alleviated intestinal barrier damage (Fig. 4E). These findings suggest that the Metrnl gene may hold therapeutic potential in the treatment of endotoxin-induced intestinal barrier damage.

### Circulating Metrnl levels increase with intestinal barrier damage

**Fig. 5:**
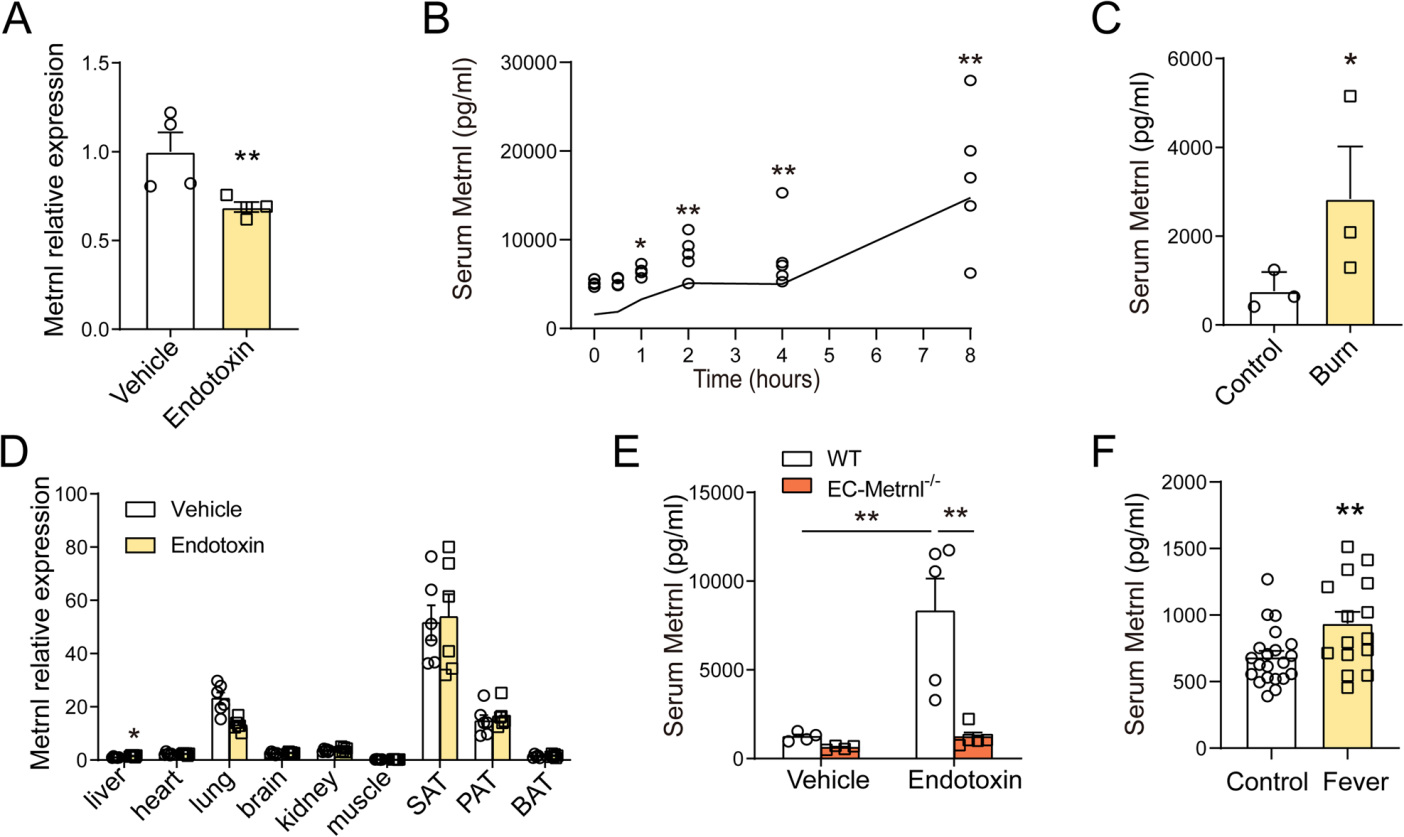
Blood Metrnl levels increase during intestinal barrier dysfunction. **A** Metrnl expression in the intestine under endotoxin stimulation. *n* = 4. **B** Circulating Metrnl levels in response to endotoxin stimulation. *n* = 4 for the 0-hour group and *n* = 5 for the 0.5, 1, 2, 4, and 8-hour groups. **C** Serum Metrnl levels in response to burn stimulation. *n* = 3. **D** Metrnl expression in various tissues under endotoxin stimulation. *n* = 6. **E** Blood Metrnl levels in Metrnl endothelium-specific knockout mice treated with or without endotoxin. *n* = 4. **F** Blood Metrnl levels in individuals with or without fever. *n* = 21 for Control group and *n* = 15 for Fever group. SAT, inguinal subcutaneous adipose tissue; PAT, periepididymal adipose tissue; BAT, interscapular brown adipose tissue. *P < 0.05; **P < 0.01, compared with corresponding controls.

Given the critical role of Metrnl in maintaining intestinal barrier function, it seems reasonable to hypothesize that Metrnl expression in the intestinal epithelium would be upregulated in response to compromised intestinal barrier function. However, our findings revealed a downregulation of Metrnl expression under conditions of endotoxin-induced intestinal barrier damage (Fig. 5A; Suppl. Fig. 4). Since Metrnl is a secreted protein, it is possible to enhance its function in intestine by increasing circulating Metrnl levels. To investigate the relationship between circulating Metrnl levels and intestinal barrier damage, we measured blood Metrnl concentration in animal models with impaired intestinal barrier function induced by administration of endotoxin or severe burns. Our findings revealed a significant increase in blood Metrnl levels after endotoxin injection (Fig. 5B). An increase in circulating Metrnl levels was also observed 10 hours post severe burn (Fig. 5C).

To investigate the source of increased blood Metrnl levels in an endotoxin-induced intestinal barrier dysfunction model, we conducted a comprehensive examination of Metrnl expression in major tissues and organs, including the liver, heart, lung, brain, kidney, muscle, white and brown adipose tissues. However, with the exception of a slight increase (1.5-fold) in the liver, most tissues did not demonstrate a statistically significant increase in Metrnl expression (Fig. 5D).

Our previous study suggested that Metrnl is primarily secreted by vascular endothelium [25]. To confirm this under endotoxin stimulation, a vascular endothelium-specific knockout mouse model (EC-Metrnl^−/−^) was employed. The results demonstrated that targeted deletion of endothelial Metrnl significantly reduced the endotoxin-induced increase in circulating Metrnl levels, although it did not completely abolish it (Fig. 5E). These findings provide further evidence supporting the hypothesis that the upregulation of Metrnl in response to endotoxin is predominantly derived from vascular endothelium.

Fever is primarily caused by systemic inflammation, which can lead to dysfunction of the intestinal barrier [26]. To investigate the association between Metrnl and intestinal barrier damage in humans, blood Metrnl levels were measured in patients with a fever higher than 38 °C (see Suppl. Table 3 for patient characteristics). The results showed an increase in circulating Metrnl in these patients (Fig. 5F), implying that Metrnl may play a protective role against intestinal barrier dysfunction in clinical settings.

### Metrnl recombinant protein alleviated endotoxin-induced intestinal barrier impairment

**Fig. 6:**
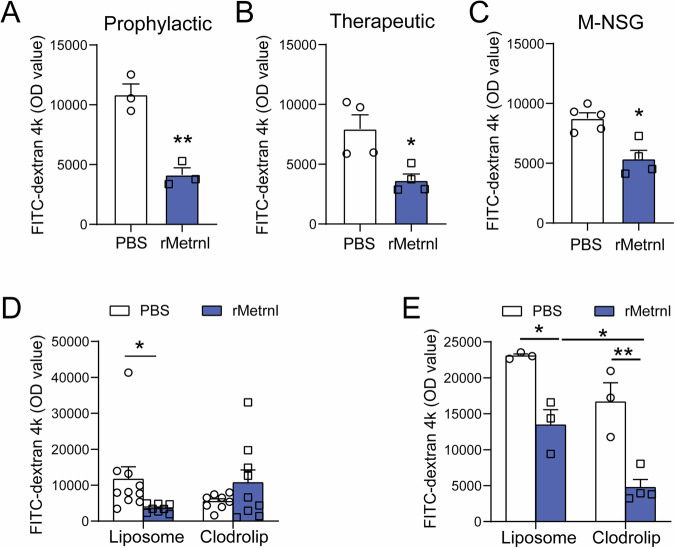
The recombinant Metrnl protein enhances intestinal barrier function independently of T, B, and NK cells. **A** Prophylactic administration of Metrnl recombinant protein prevents endotoxin-induced intestinal barrier dysfunction. *n* = 3. **B** Therapeutic administration of Metrnl recombinant protein ameliorates endotoxin-induced intestinal barrier dysfunction. *n* = 4. **C** Protective effects of Metrnl recombinant protein on intestinal barrier function in immune-deficient mice (M-NSG) lacking T cells, B cells, and NK cells. *n* = 5 for PBS group and *n* = 4 for rMetrnl group. **D** The deprivation of macrophages has cancelled the improvement in endotoxin-induced intestinal barrier function damage. *n* = 10 for PBS+liposome, *n* = 8 for PBS+Clodrolip, *n* = 8 for rMetrnl+liposome, *n* = 9 for rMetrnl+Clodrolip. **E** Role of macrophages in the Metrnl-regulated intestinal barrier dysfunction induced by severe burn. Macrophages were depleted using clodronate liposomes three days prior to burn. *n* = 3. *P < 0.05; **P < 0.01, compared with corresponding controls.

To further evaluate the therapeutic potential of Metrnl recombinant protein for endotoxin-induced intestinal barrier impairment, C57 mice were administered with Metrnl protein at a dose of 4 μg/mouse either 30 minutes prior to or 60 minutes after intraperitoneal injection of endotoxin. The results demonstrated that both prophylactic and therapeutic administration of Metrnl protein significantly improved intestinal barrier impairment (Fig. 6A, B), suggesting that Metrnl protein could serve as a promising therapeutic agent for treating intestinal barrier damage.

### Deficiency of T cells, B cells, NK cells **or macrophages** did not completely abrogate the alleviation of intestinal barrier impairment by Metrnl recombinant protein

The effect of immune function on the therapeutic efficacy of Metrnl recombinant protein on endotoxin-induced intestinal barrier function was evaluated using a M-NSG mouse model lacking T cells, B cells, and NK cells. The results showed that the administration of Metrnl recombinant protein effectively improved intestinal barrier damage induced by endotoxin, even in the absence of T cells, B cells, and NK cells (Fig. 6C). These findings suggest that Metrnl recombinant protein may regulate intestinal barrier function through mechanisms independent of these immune cells.

To investigate the potential role of macrophages in regulating intestinal barrier function by Metrnl, chlorophospholipid was utilized in vivo to remove macrophages. Mouse models with depleted macrophages were subjected to endotoxin-induced or severe burn-induced intestinal barrier injury. Notably, absence of macrophages cancelled the improvement effect of Metrnl on endotoxin-induced intestinal barrier damage (Fig. 6D). However, in the case of severe burn-induced intestinal barrier injury, the Metrnl recombinant protein still facilitated the improvement, despite the depletion of macrophages (Fig. 6E). These findings suggest that macrophages are not necessary for the regulation of intestinal barrier function by Metrnl.

### Metrnl deficiency enhances the NFκB signaling pathway in enterotypes

**Fig. 7:**
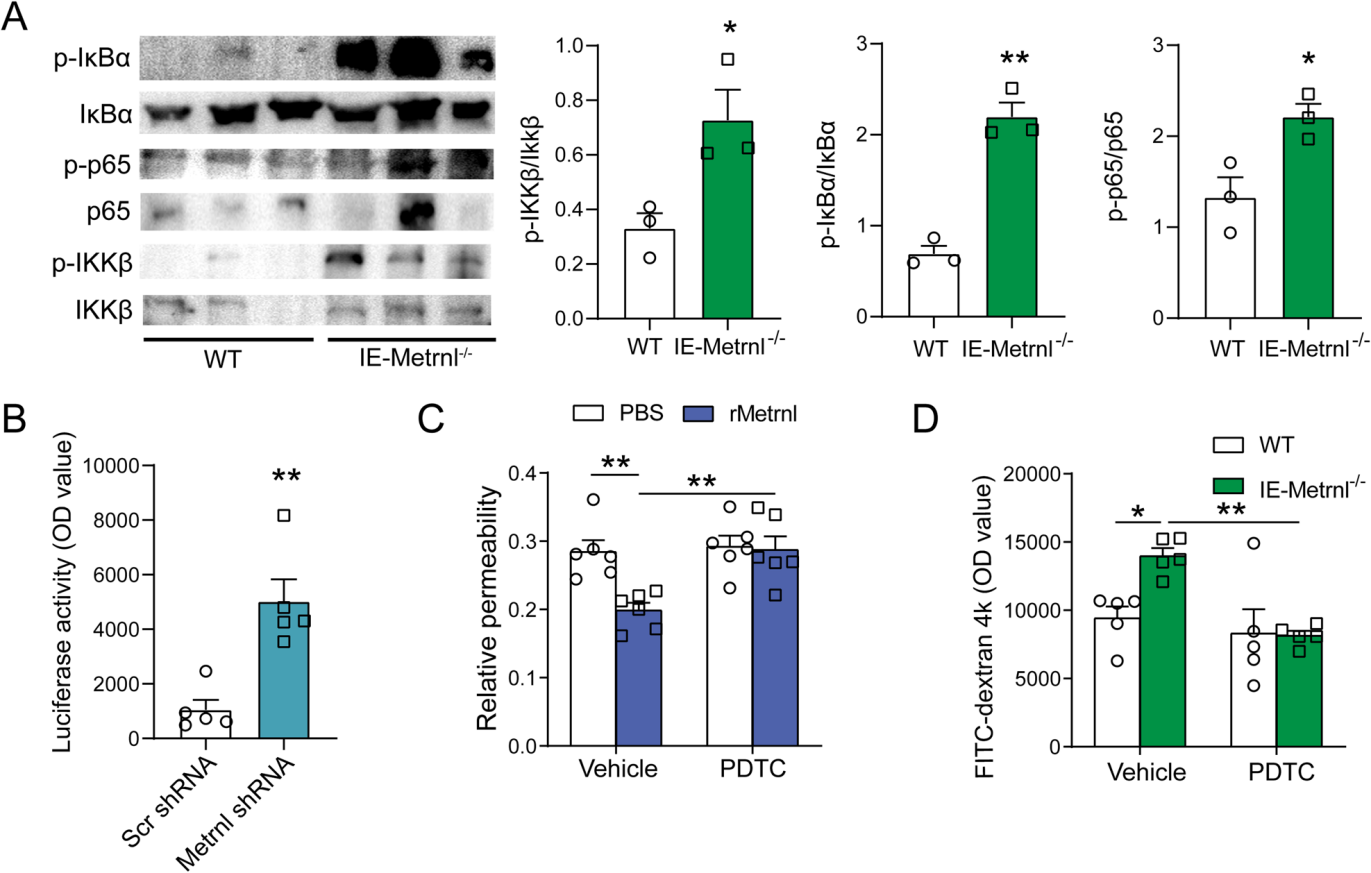
NFκB signaling pathway mediated Metrnl deficiency attenuates intestinal barrier dysfunction. **A** Western blot analysis of NFκB signaling pathway-related molecules, including phosphorylation of IKKβ, IκBα, and p65 in Metrnl intestinal-specific knockout (IE-Metrnl^−/−^) and wild type mice (WT) under endotoxin treatment. *n* = 3. **B** NFκB transcriptional activity in Caco2 cells knocked down with Metrnl shRNA or Scramble shRNA (Scr shRNA) under endotoxin stimulation. *n* = 5. **C** The effect of pyrrolidine dithiocarbamate (PDTC), an inhibitor of the NFκB signaling pathway, on the permeability of Caco2 monolayer treated with or without Metrnl recombinant protein under endotoxin stimulation. Relative permeability is defined as the ratio of the concentration of FITC-dextran in the upper chamber to the concentration of FITC-dextran in the bottom chamber. *n* = 6. **D** The effect of PDTC on intestinal barrier function of IE-Metrnl^−/−^ and wild type mice under endotoxin treatment. *n* = 6. *P < 0.05; **P < 0.01, compared with corresponding controls.

In order to further investigate the mechanisms underlying the regulation of tight junctions in enterotypes by Metrnl, the role of NFκB was examined, as it is known to regulate enterocyte tight junctions. The result shows that phosphorylation of p65, IκB, and IKKβ was also enhanced in intestinal tissue from IE-Metrnl^−/−^ mice compared to wild-type mice (Fig. 7A). To confirm that Metrnl deficiency enhances NFκB transcriptional activity, pNFκB-luc plasmids were transfected into Caco2 cells with or without Metrnl deficiency, and luciferase activity was measured as an indicator of transcriptional activity. The results showed that NFκB transcriptional activity was significantly increased in the Metrnl shRNA group compared to the scrambled shRNA group (Fig. 7B), indicating that Metrnl deficiency promotes NFκB transcriptional activity.

### Inhibition of the NFκB signaling pathway abrogated the intestinal barrier impairment caused by Metrnl deficiency

To investigate the potential role of the NFκB signaling pathway in Metrnl deficiency-induced intestinal barrier damage, PDTC, an inhibitor of the NFκB signaling pathway, was employed. Treatment with PDTC completely abrogated the improvement in FITC-dextran 4k permeability conferred by Metrnl recombinant protein in differentiated Caco2 cells treated with endotoxin (Fig. 7C). Additionally, oral administration of a low dose of PDTC eliminated the enhancement of intestinal barrier damage induced by Metrnl deficiency (Fig. 7D), providing further evidence for the involvement of the NFκB signaling pathway in this process.

### Intestinal epithelium-specific overexpression of Metrnl alleviates DSS-induced colitis

**Fig. 8:**
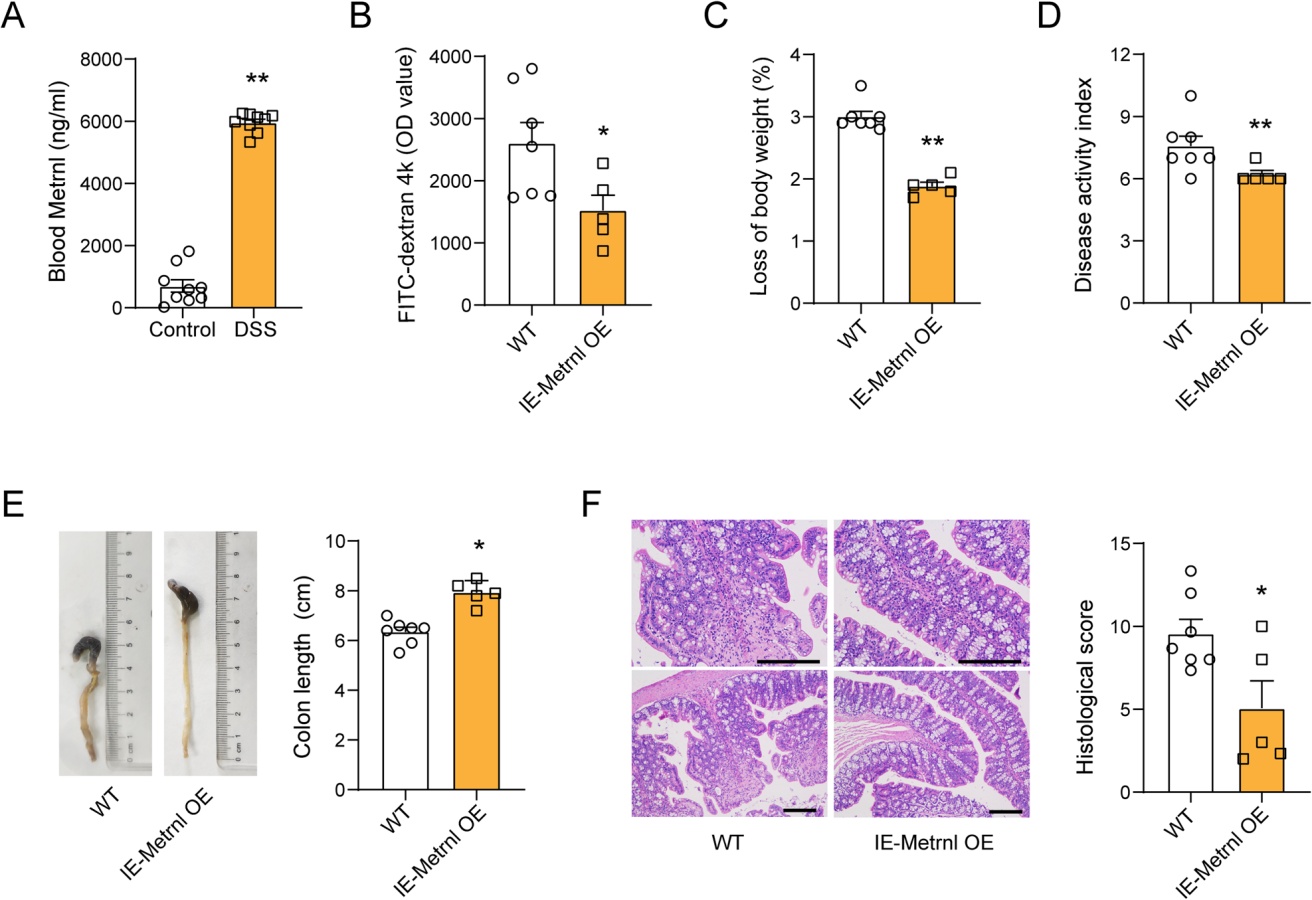
Enterocyte metrnl overexpression alleviates DSS-induced colitis. **A** Blood Metrnl levels increased in mice with dextran sodium sulfate (DSS)-induced colitis. *n* = 9. **B** Enterocyte-specific overexpression of Metrnl alleviated endotoxin-induced intestinal barrier injury. *n* = 7 for wild type group and *n*=5 for IE-Metrnl OE group. **C** Enterocyte-specific overexpression of Metrnl mitigated body weight loss in mice with DSS-induced colitis. *n* = 7 for WT group and *n* = 5 for IE-Metrnl OE group. **D** The disease activity index assessment demonstrated that DSS-induced colitis symptoms were alleviated in IE-Metrnl OE mice. *n* = 7 for WT group and *n* = 5 for IE-Metrnl OE group. **E** Enterocyte-expressed Metrnl prevented colon shortening induced by colitis in mice. *n* = 7 for WT group and *n* = 5 for IE-Metrnl OE group. **F** Histological scores indicated improved intestinal inflammation in mice with enterocyte-specific Metrnl overexpression. *n* = 7 for WT group and *n* = 5 for IE-Metrnl OE group. *P < 0.05 and **P < 0.01, compared with corresponding controls.

Intestinal barrier function is closely related to the occurrence and development of colitis. We observed a significant increase in the concentration of Metrnl in the blood of DSS-induced colitis (Fig. 8A). To further explore the potential protective effects of intestinal epithelial Metrnl on colitis, we induced colitis in IE-Metrnl OE and wild-type mice with 2.5% DSS and evaluated the impairment of intestinal barrier function and severity of colitis one week later. The results showed that DSS-induced intestinal barrier damage was significantly alleviated (Fig. 8B). Moreover, colitis-induced weight loss and disease symptoms were reduced in IE-Metrnl OE mice (Fig. 8C, D). As detected by pathological examination, both colon shortening and colon tissue damage were significantly reduced (Fig. 8E, F). These results suggest that Metrnl in the intestinal epithelium can significantly improve the occurrence and development of colitis.

## Discussion

The present study provides new insights into the critical role of Metrnl in maintaining the integrity of the intestinal barrier. Metrnl is abundantly expressed in enterocytes, and its circulating levels increase in response to endotoxin- and severe burn-induced damage to the intestinal barrier. Global or enterocyte-specific knockout of Metrnl leads to the deterioration of endotoxin- and burn-induced intestinal barrier dysfunction, while overexpression of Metrnl in the intestinal epithelium or administration of Metrnl recombinant protein improves endotoxin-induced intestinal barrier impairment. Further results reveal that Metrnl deficiency increases inflammatory response, disrupts tight junctions, upregulates phosphorylation of MLC and expression of MLCK in IE-Metrnl^−/−^ mice, and increases transmembrane potential and permeability for FITC-Dextran 4k in differentiated Caco2 cell monolayers. Additional experiments indicate that Metrnl deficiency enhances the IKKβ/IκBα/NFκB signaling pathway. Inhibition of NFκB with a small molecule inhibitor eliminates Metrnl deficiency-induced intestinal barrier impairment both in vivo and in vitro (Graphical abstract). Further works showed that enterocyte Metrnl improved DSS-induced colitis. These findings demonstrate the importance of Metrnl in regulating the integrity of the intestinal barrier and provide a potentially promising therapeutic target for the treatment of intestinal barrier-related diseases.

Previous studies have reported that Metrnl is highly expressed in inflammatory tissues and involved in the inflammatory response [23, 24, 27]. Our prior research has also revealed that Metrnl can regulate adipose and vascular endothelial inflammation, contributing to the development of insulin resistance and atherosclerosis [2, 25], meanwhile, Metrnl deficiency increases mortality in endotoxin-induced systemic inflammatory response syndrome [24]. Based on these findings, it was hypothesized that Metrnl might regulate intestinal barrier function through the modulation of inflammation. Indeed, Metrnl deficiency has led to notable elevations in IL1β in the bloodstream, as well as IL6 expression in the intestinal tissue. The exacerbation of endotoxin-induced intestinal barrier dysfunction attributable to Metrnl deficiency was mitigated by macrophage deprivation, but not by the absence of T cells, B cells, or NK cells. Furthermore, the lack of macrophages did not negate Metrnl’s beneficial effect on burn-induced intestinal barrier dysfunction. These observations suggest that Metrnl regulates intestinal barrier function via both inflammation-dependent and independent pathways.

Apoptosis and shedding of enterocytes are recognized as contributors to intestinal barrier dysfunction in various pathological conditions, including endotoxemia [16, 17, 21]. However, the role of Metrnl in regulating apoptosis in enterotypes and its association with intestinal barrier function are not fully understood. The study investigated the effect of Metrnl on enterocyte apoptosis in IE-Metrnl mice and Caco2 cells treated with endotoxin by detecting TUNEL, caspase 3 activity, and FITC-annexin V positive cells. Surprisingly, the results did not reveal a significant increase in apoptosis in intestinal epithelial cells deficient in Metrnl, even under sub-lethal doses of endotoxin (5 mg/kg). These findings are consistent with our recent study, which showed that Metrnl deficiency promotes cell survival and reduces apoptosis of intestinal epithelial Caco2 cells treated with 5-fluorouracil [28]. Therefore, it appears that Metrnl does not regulate intestinal barrier function through apoptosis of enterocytes in endotoxemia model, although its role in apoptosis regulation under other pathological conditions, such as DSS-induced colitis, remains to be elucidated.

NFκB signal pathway plays a crucial role in maintaining intestinal barrier function and is involved in the impairment of the intestinal barrier induced by endotoxin, inflammation, ischemia-reperfusion, burn, and other pathological conditions [29]. Activation of NFκB can promote cell survival but also disrupt tight junctions via the MLCK-MLC pathway [25]. On the other hand, inhibition of NFκB can prevent inflammation-induced loosening of tight junctions but increase susceptibility to apoptosis [29]. Silencing Metrnl resulted in increased phosphorylation of p65, IκB, and IKKβ, and transcriptional activation of NFκB. Blocking the NFκB signaling pathway abolished Metrnl-regulated intestinal barrier impairment, demonstrating the pivotal role of the NFκB signaling pathway in Metrnl-mediated intestinal function.

The relationship between intestinal barrier function and colitis has been well established [14]. Previous research has shown that knocking out Metrnl in intestinal epithelial cells can exacerbate experimental colitis [13], but the underlying mechanism remains unclear. The therapeutic potential of locally administered Metrnl for treating colitis also remains unconfirmed. This study suggests that Metrnl may improve colitis not only by inhibiting inflammation but also by protecting the intestinal barrier function. It also provides evidence supporting the use of Metrnl protein for local treatment as a promising strategy for treating colitis. However, our study did not examine Metrnl’s effects on barrier permeability in specific intestinal segments. Future studies focusing on colonic barrier function are needed to better understand its therapeutic potential. Given that DSS-induced colitis represents an experimental model with distinct differences from human disease pathology, future validation through clinical studies or more physiologically relevant disease models will be essential to establish the translational relevance of our findings.

In conclusion, this study demonstrates that Metrnl, which is highly expressed in enterocytes and increases in the bloodstream during intestinal barrier damage, plays a crucial role in maintaining the integrity of the intestinal barrier by protecting against tight junction disruption from endotoxin and burn via inhibition of inflammation and the IKKβ/IκBα/NFκB/MLCK/MLC signaling pathway. Both overexpression of Metrnl and administration of Metrnl protein were found to improve intestinal barrier dysfunction. These findings highlight the potential therapeutic value of Metrnl in maintaining intestinal barrier function and developing novel therapeutic strategies for conditions associated with intestinal barrier dysfunction.

## Materials and methods

### Mouse models

All animal experiments were carried out in accordance with the National Institute of Health Guide for the Care and Use of Laboratory Animals and were approved by the Institutional Animal Care and Use Committee of the Naval Medical University. Mice with a global knockout of Metrnl were generated by crossing floxed Metrnl mice (Metrnl^loxp/loxp^) with EIIa-Cre mice. In addition, we generated an intestinal epithelial-specific knockout (IE-Metrnl^−/−^) and a vascular endothelium-specific knockout mouse (EC-Metrnl^−/−^) by crossing Metrnl^loxp/loxp^ mice with Villin-Cre or Tie2-Cre mice, respectively, as previously described [3, 8, 25].

Conditional overexpression of Metrnl was achieved by microinjecting guide RNA, Cas9 mRNA, and a homologous recombination vector containing a CAG-LoxP-stop-LoxP-Metrnl-3*flag-WPRE-PA fragment flanked by 3.3 kb homologous arms of the Rosa26 gene insertion site into C57 mouse zygotes. M-NSG and Vil-cre mice were obtained from the Shanghai Research Center for Model Organisms and maintained on a 12-hour light-dark cycle with free access to food and water. Both male and female animals, aged between 8 and 12 weeks, were used in the experiment. For animal studies, we determined sample sizes based on our previous experience with similar experimental models, established in the field, and ethical principles. To minimize potential bias, investigators were blinded to group allocation during the experimental procedures.

### Assessment of intestinal permeability

Fluorescein-isothiocyanate (FITC)-conjugated dextran 4k was dissolved in PBS at a concentration of 22 mg/mL. The animals were fasted overnight and orally administered FITC-dextran 4k at a dose of 20 mL/kg via gavage. Blood was collected from the tail vein 5 or10 hours after gavage. For the burn model, mice were anesthetized using isoflurane and then given a full-thickness burn covering 30% of their total body surface area. Ten hours post-burn, the animals were anesthetized again, and a 5 cm segment from the distal ileum was isolated and injected with FITC-dextran. After 30 minutes, blood samples were collected from the tail vein. After an additional hour, the sample was centrifuged, and the resulting supernatant was diluted by 50%. The fluorescence intensity of the supernatant was measured using a fluorescence enzyme analyzer with excitation and emission wavelengths of 485 nm and 525 nm, respectively.

### Detection of intestinal epithelium barrier in vitro

To evaluate the barrier function of intestinal epithelial cells, Caco-2 cells were cultured in Tranwell chambers with 0.4-micrometer pores (Corning Incorporated, Kennebunk, ME, USA) for two weeks to facilitate proper barrier formation. Changes in trans-epithelial electrical resistance on both the apical and basolateral sides of the chamber were measured to confirm barrier formation. Subsequently, endotoxin (Sigma-Aldrich, MO, USA) was added when the resistance exceeded 800 ohms. Barrier permeability was evaluated by introducing FITC-dextran 4k to the chamber and measuring the fluorescence intensity of FITC in the culture medium beneath the chamber.

### Immunohistochemistry

Intestinal tissue specimens are obtained from sections of the ileum adjacent to the cecum, unless stated otherwise. Immunohistochemistry was carried out following established protocols [25]. Briefly, tissue sections of 4 μm were subject to microwave-based antigen retrieval in citrate buffer, followed by blocking of endogenous peroxidase activity. Primary antibodies against Metrnl (Sigma-Aldrich, Stockholm, Sweden) were incubated with the sections overnight at 4 °C. Biotinylated secondary antibodies (Abcam, Cambridge, UK) were subsequently added to the sections, followed by avidin-conjugated horseradish peroxidase. Examination of the sections was carried out using a Leica DM LB2 microscope.

### Electron microscopy sample preparation

The samples were fixed with a combination of 2.5% glutaraldehyde and 1% osmium tetroxide, then dehydrated using a graded ethanol series, embedded in epoxy resin, and subsequently sectioned into ultrathin slices (60–80 nm). These sections were subsequently stained with 2% uranyl acetate and 2.6% lead citrate before examination under an electron microscope.

### Real time PCR

Total RNA was extracted from tissues or cells using Trizol Reagent (Invitrogen, Carlsbad, CA, USA) following the manufacturer’s instructions. Real-time PCR was performed using a FastStart Universal SYBR Green Master (ROX) (Roche, Basel, Switzerland) and an ABI7500 Real-time PCR system. The expression levels of Metrnl were determined using the 2^(−ΔΔCt) method, with GAPDH as the internal control. The data were presented as fold change. The primers used in the study are listed in Suppl. Table 1.

### Western blotting

The Western blot analysis was conducted as previously described [25]. Briefly, tissue samples were harvested and homogenized in protein extraction buffer (Beyotime, Shanghai, China). An equal amount of protein was separated using SDS-PAGE and then transferred onto a polyvinylidene difluoride (PVDF) (Millipore Sigma, Burlington, MA, USA). After blocking with skim milk, the primary antibody was added and incubated overnight at 4 °C. Then, infrared-dyes-conjugated secondary antibodies (Abcam, Cambridge, UK) were added and incubated with the membranes for 30 minutes. For phosphorylated protein detection, parallel gels with identical samples were run to ensure more accurate and reliable quantification of both phosphorylated and total protein levels. Images were captured using an Odyssey infrared imaging system (Li-Cor Bioscience, Lincoln, NE, USA). The primary antibody information is listed in Suppl. Table 2.

### ELISA measurements of Metrnl

The concentrations of Metrnl were determined using a Mouse or Human Meteorin-like DuoSet ELISA kit (R&D, Minneapolis, MN, USA), following the manufacturer’s instructions and consistent with previous studies [25].

### Apoptosis detection

Caco2 cells were obtained from the Cell Bank of the Chinese Academy of Sciences (Shanghai, China). The cells were authenticated by STR profiling and tested negative for mycoplasma contamination. Caco2 cells were exposed to endotoxin for a duration of 24 hours and thereafter subjected to double-labeling using Annexin V-FITC and PI (Sigma-Aldrich, MO, USA). Flow cytometry was performed to detect apoptosis. Caspase 3 activity was quantified in Caco2 cells or intestinal tissue using a Caspase 3 activity detection kit (Beyotime, Shanghai, China) in accordance with manufacturer’s instructions. Furthermore, TUNEL staining was conducted on intestinal tissue utilizing established protocols, which included polyformaldehyde fixation, embedding, 4 μm sectioning, and TUNEL staining (Beyotime, Shanghai, China). The quantity of TUNEL-positive cells was evaluated in four arbitrarily selected fields of view.

### Virus-mediated knockdown of Metrnl

The lentivirus to knock down human Metrnl was constructed by Shanghai Bio-Link Company (Shanghai, China). To construct human Metrnl shRNA lentivirus, four targeting sequences were designed, which included 5’-GCTTCCAGTACGAGCTGGTTA-3’, 5’-AGAACTGAGACTGCTGGTA-3’, 5’-GCCGATTGGAAATGCTGTAAA-3’, and 5’-CAGGTGCTCTCATCGTTAACC-3’. The targeting sequence with the highest knockdown efficiency, which was the last one, was used in the present study. Caco2 intestinal epithelial cells were used for the study. These cells were purchased from ATCC and cultured in DMEM medium (Sigma-Aldrich, MO, USA) supplemented with 10% fetal bovine serum (Sigma-Aldrich, MO, USA). The cells were transfected at a multiplicity of infection of 10.

### Human plasma collection

A total of 36 patients were enrolled from the Fifth Medical Center of the People’s Liberation Army (PLA) General Hospital. Of these, 21 patients had no fever, while 15 patients had a fever. Body temperature was measured using a glass mercury thermometer placed in the mouth for five minutes. A temperature above 38 °C for three consecutive days was defined as a fever. Venous blood samples were collected for Metrnl assays. The study was conducted in compliance with the Declaration of Helsinki and was approved by the Medical Ethical Committee of the Fifth Medical Center of the PLA General Hospital (HZKY-PJ-2020-31). Written consent was obtained from all participants. The baseline information for the two groups of patients was provided in Suppl. Table 2.

### Establishment and evaluation of intestinal inflammation model

Male IE-Metrnl Tg mice and their littermate control mice, 10 weeks old, were given free access to water containing 2.5% DSS for 7 consecutive days, with daily refreshment. On the 8th day of the experiment, the mice were weighed and their feces were collected for observation of fecal morphology. The disease activity index was determined by combining the scores of (i) body weight loss, (ii) stool inconsistency, and (iii) presence of blood in the stool, as we described elsewhere [13]. The mice were sacrificed by an overdose of pentobarbital, and the colon was removed for length measurement and histological examination. Histological scoring was performed as previously described [13], which included inflammation (0-3), extent (0-3), regeneration (0-4), crypt damage (0-4), and percent involvement (1-4). Slides were coded and assessed by investigators who were blinded to the experimental groups to ensure unbiased scoring of morphological changes.

### Statistical analysis

Sample sizes are determined based on our prior experience with similar experimental models, established practices in the field, and ethical considerations. All sample sizes are reported as exact numbers in the respective figure legends. In animal studies, animals are randomly allocated to experimental groups, except in Metrnl transgenic studies where same-sex littermate controls were utilized to minimize biological variation. All experimental groups underwent simultaneous processing and analysis to maintain consistent treatment conditions. All data points from our experiments were included in the analyses with no exclusions.

All data are expressed as the mean ± standard deviation error (SEM). Statistical analysis was performed using SPSS statistical software version 25.0 (IBM Corp, Chicago, IL, USA). Normality was tested with the Kolmogorov-Smirnov test. If no significant differences in variance between groups (F test) were found and normality was confirmed, differences were evaluated using two-tailed Student’s t-test. Otherwise, the nonparametric Mann-Whitney U test was used. For the analysis of four groups, a two-way ANOVA was used, followed by Tukey’s multiple comparisons test. If the data did not conform to a normal distribution, a log transformation was applied prior to the statistical analysis. P-values less than 0.05 were considered statistically significant.

## Supplementary information

Supplementary information

Supporting material

## Data Availability

Original data are available upon request.

## References

[1] Miao ZW, Hu WJ, Li ZY, Miao CY. Involvement of the secreted protein Metrnl in human diseases. Acta Pharmacol Sin. 2020;41:1525–30. 32999412 10.1038/s41401-020-00529-9 PMC7921639

[2] Li ZY, Song J, Zheng SL, Fan MB, Guan YF, Qu Y, et al. Adipocyte Metrnl Antagonizes Insulin Resistance Through PPARgamma Signaling. Diabetes. 2015;64:4011–22. 26307585 10.2337/db15-0274

[3] Qi Q, Hu WJ, Zheng SL, Zhang SL, Le YY, Li ZY, et al. Metrnl deficiency decreases blood HDL cholesterol and increases blood triglyceride. Acta Pharmacol Sin. 2020;41:1568–75. 32265491 10.1038/s41401-020-0368-8 PMC7921638

[4] Jorgensen JR, Fransson A, Fjord-Larsen L, Thompson LH, Houchins JP, Andrade N, et al. Cometin is a novel neurotrophic factor that promotes neurite outgrowth and neuroblast migration in vitro and supports survival of spiral ganglion neurons in vivo. Exp Neurol. 2012;233:172–81. 21985865 10.1016/j.expneurol.2011.09.027

[5] Baht GS, Bareja A, Lee DE, Rao RR, Huang R, Huebner JL, et al. Meteorin-like facilitates skeletal muscle repair through a Stat3/IGF-1 mechanism. Nat Metab. 2020;2:278–89. 32694780 10.1038/s42255-020-0184-y PMC7504545

[6] Reboll MR, Klede S, Taft MH, Cai CL, Field LJ, Lavine KJ, et al. Meteorin-like promotes heart repair through endothelial KIT receptor tyrosine kinase. Science. 2022;376:1343–7. 35709278 10.1126/science.abn3027 PMC9838878

[7] Hu C, Zhang X, Song P, Yuan YP, Kong CY, Wu HM, et al. Meteorin-like protein attenuates doxorubicin-induced cardiotoxicity via activating cAMP/PKA/SIRT1 pathway. Redox Biol. 2020;37:101747. 33045622 10.1016/j.redox.2020.101747 PMC7558217

[8] Li ZY, Fan MB, Zhang SL, Qu Y, Zheng SL, Song J, et al. Intestinal Metrnl released into the gut lumen acts as a local regulator for gut antimicrobial peptides. Acta Pharmacol Sin. 2016;37:1458–66. 27546006 10.1038/aps.2016.70 PMC5099411

[9] Citi S. Intestinal barriers protect against disease. Science. 2018;359:1097–8. 29590026 10.1126/science.aat0835

[10] Camilleri M. Leaky gut: mechanisms, measurement and clinical implications in humans. Gut. 2019;68:1516–26. 31076401 10.1136/gutjnl-2019-318427 PMC6790068

[11] Yoseph BP, Klingensmith NJ, Liang Z, Breed ER, Burd EM, Mittal R, et al. Mechanisms of Intestinal Barrier Dysfunction in Sepsis. Shock. 2016;46:52–9. 27299587 10.1097/SHK.0000000000000565 PMC4910519

[12] Mittal R, Coopersmith CM. Redefining the gut as the motor of critical illness. Trends Mol Med. 2014;20:214–23. 24055446 10.1016/j.molmed.2013.08.004 PMC3959633

[13] Zhang SL, Li ZY, Wang DS, Xu TY, Fan MB, Cheng MH, et al. Aggravated ulcerative colitis caused by intestinal Metrnl deficiency is associated with reduced autophagy in epithelial cells. Acta Pharmacol Sin. 2020;41:763–70. 31949292 10.1038/s41401-019-0343-4 PMC7471395

[14] Odenwald MA, Turner JR. The intestinal epithelial barrier: a therapeutic target? Nat Rev Gastroenterol Hepatol. 2017;14:9–21. 27848962 10.1038/nrgastro.2016.169 PMC5554468

[15] Peterson LW, Artis D. Intestinal epithelial cells: regulators of barrier function and immune homeostasis. Nat Rev Immunol. 2014;14:141–53. 24566914 10.1038/nri3608

[16] Coopersmith CM, Chang KC, Swanson PE, Tinsley KW, Stromberg PE, Buchman TG, et al. Overexpression of Bcl-2 in the intestinal epithelium improves survival in septic mice. Crit Care Med. 2002;30:195–201. 11902262 10.1097/00003246-200201000-00028

[17] Coopersmith CM, Stromberg PE, Dunne WM, Davis CG, Amiot DM, 2nd. Inhibition of intestinal epithelial apoptosis and survival in a murine model of pneumonia-induced sepsis. JAMA. 2002;287:1716–21. 11926897 10.1001/jama.287.13.1716

[18] Chelakkot C, Ghim J, Ryu SH. Mechanisms regulating intestinal barrier integrity and its pathological implications. Exp Mol Med. 2018;50:1–9. 30115904 10.1038/s12276-018-0126-x PMC6095905

[19] Graham WV, He W, Marchiando AM, Zha J, Singh G, Li HS, et al. Intracellular MLCK1 diversion reverses barrier loss to restore mucosal homeostasis. Nat Med. 2019;25:690–700. 30936544 10.1038/s41591-019-0393-7 PMC6461392

[20] Jin Y, Blikslager AT. The Regulation of Intestinal Mucosal Barrier by Myosin Light Chain Kinase/Rho Kinases. Int J Mol Sci. 2020;21:3550. 32443411 10.3390/ijms21103550 PMC7278945

[21] Su L, Nalle SC, Shen L, Turner ES, Singh G, Breskin LA, et al. TNFR2 activates MLCK-dependent tight junction dysregulation to cause apoptosis-mediated barrier loss and experimental colitis. Gastroenterology. 2013;145:407–15. 23619146 10.1053/j.gastro.2013.04.011 PMC3722284

[22] He W, Wang Y, Wang P, Wang F. Intestinal barrier dysfunction in severe burn injury. Burns Trauma. 2019;7:24. 31372365 10.1186/s41038-019-0162-3 PMC6659221

[23] Ushach I, Burkhardt AM, Martinez C, Hevezi PA, Gerber PA, Buhren BA, et al. METEORIN-LIKE is a cytokine associated with barrier tissues and alternatively activated macrophages. Clin Immunol. 2015;156:119–27. 25486603 10.1016/j.clim.2014.11.006 PMC4336607

[24] Ushach I, Arrevillaga-Boni G, Heller GN, Pone E, Hernandez-Ruiz M, Catalan-Dibene J, et al. Meteorin-like/Meteorin-beta Is a Novel Immunoregulatory Cytokine Associated with Inflammation. J Immunol. 2018;201:3669–76. 30464051 10.4049/jimmunol.1800435 PMC6394858

[25] Zheng S, Li Z, Song J, Wang P, Xu J, Hu W, et al. Endothelial METRNL determines circulating METRNL level and maintains endothelial function against atherosclerosis. Acta Pharm Sin B. 2023;13:1568–87. 37139425 10.1016/j.apsb.2022.12.008 PMC10149902

[26] Barichello T, Generoso JS, Singer M, Dal-Pizzol F. Biomarkers for sepsis: more than just fever and leukocytosis-a narrative review. Crit Care. 2022;26:14. 34991675 10.1186/s13054-021-03862-5 PMC8740483

[27] Zuo L, Ge S, Ge Y, Li J, Zhu B, Zhang Z, et al. The Adipokine Metrnl Ameliorates Chronic Colitis in Il-10−/− Mice by Attenuating Mesenteric Adipose Tissue Lesions During Spontaneous Colitis. J Crohns Colitis. 2019;13:931–41. 30615095 10.1093/ecco-jcc/jjz001

[28] Luo HY, Wang X, Yu ED, Zhu LL, Yang JH, Hou YS, et al. Downregulation of Metrnl promotes development and progression of colorectal cancer *Academic*. Journal of Naval Medical University. 2023;44:222–5.

[29] Nenci A, Becker C, Wullaert A, Gareus R, van Loo G, Danese S, et al. Epithelial NEMO links innate immunity to chronic intestinal inflammation. Nature. 2007;446:557–61. 17361131 10.1038/nature05698

