# Supplementary material for: Metrnl protects intestinal barrier function by regulating tight junctions via the IKKβ/IκBα/NFκB/MLCK/MLC signaling pathway
Source: Cell Death Discov. 2025 Apr 8;11:155. doi: 10.1038/s41420-025-02457-1 (PMC11979045; doi:10.1038/s41420-025-02457-1)
Supplement: Supplementary file 1 — Supplementary information [file 41420_2025_2457_MOESM1_ESM.docx]

**Supplementary materials**

**Metrnl Protects Intestinal Barrier Function by Regulating Tight Junctions via the IKKβ/IκBα/NFκB/MLCK/MLC Signaling Pathway**

Zhi-Yong Li^1,2#^, Heng-Yu Luo^1#^, Fei Xu^1^, Yao Xu^1^, Chun-Hui Ma^2^, Sai-Long Zhang^1^, Sheng Xu^3^, Yuan-Yuan Ma^4^, Nan Li^3^, Chao-Yu Miao^1^

# ^#^The first two authors contributed equally to this work.

^1^Department of Pharmacology, Second Military Medical University/Naval Medical University, Shanghai 200433, China; ^2^Department of Pathology, Faculty of Medical Imagiology, Second Military Medical University/Naval Medical University, Shanghai 200433, China; ^3^Department of Immunology, Second Military Medical University/Naval Medical University, Shanghai 200433, China; ^4^Senior Department of Hematology, The Fifth Medical Center of People's Liberation Army(PLA), General Hospital, Beijing 100010, China.

**Correspondence**

Address correspondence to: Chao-Yu Miao, MD, PhD, and Li Zhi-Yong, MD, PhD, Department of Pharmacology, Second Military Medical University/Naval Medical University, cymiao@smmu.edu.cn and zhiyongli@smmu.edu.cn


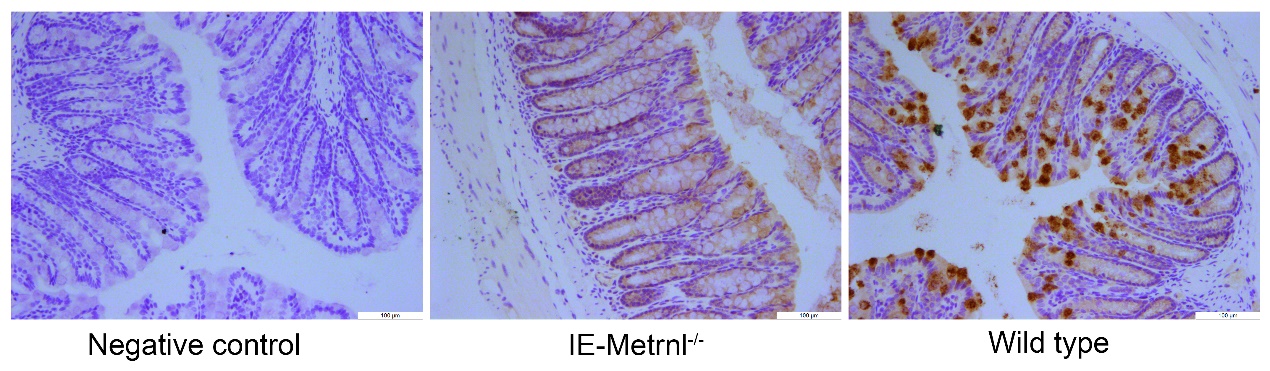


Suppl. Fig. 1: Detection of Metrnl expression in intestinal epithelium in IE-Metrnl^-/-^ and wild type mice by immunohistochemistry.


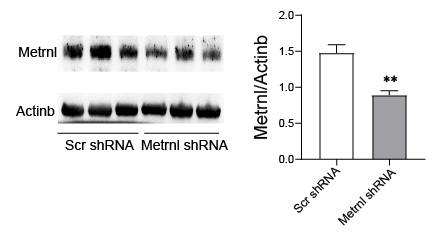


Suppl. Fig. 2: Detection of Metrnl expression in Caco2 cells after transfection of lentivirus containing Metrnl shRNA by western blot. Scr shRNA, scrambled shRNA. **, P<0.01 vs. Scr shRNA.


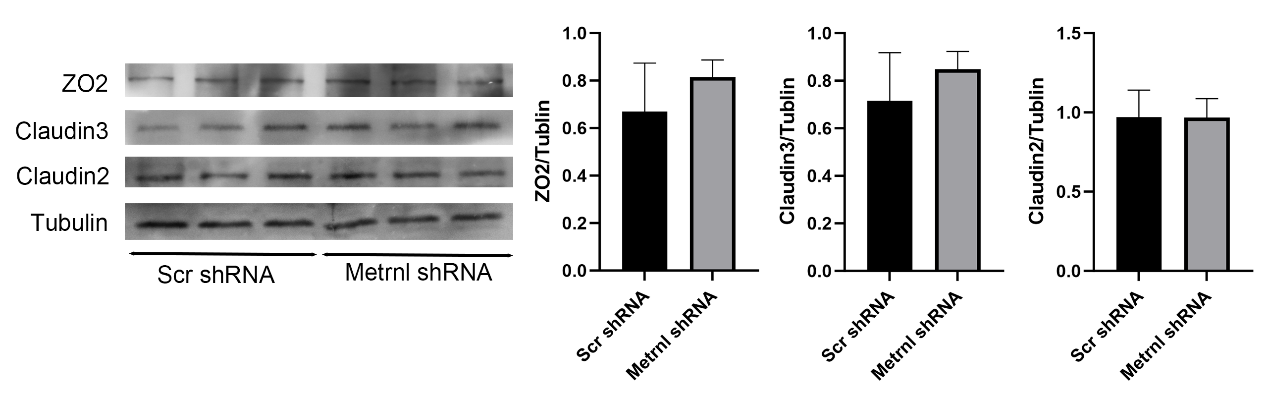


Suppl. Fig. 3: Detection of ZO2, Claudin3 and Claudin2 expression in Caco2 cells after silencing Metrnl when stimulation with endotoxin by western blot.


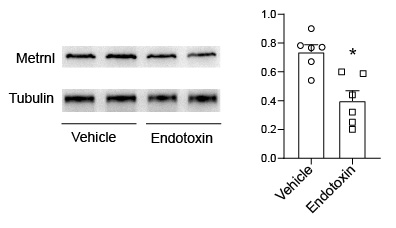


Suppl. Fig. 4: Detection of Metrnl expression in intestine after stimulation with endotoxin by western blot. *, P<0.05 vs. Vehicle.

Suppl. Table 1. Primers used in real time PCR in the study.

| Gene name | Forward**（5′–3′）** | Reward**（5′–3′）** |
| --- | --- | --- |
| Mouse Metrnl | CTGGAGCAGGGAGGCTTATTT | GGACAACAAAGTCACTGGTACAG |
| Mouse GAPDH | GTATGACTCCACTCACGGCAAA | GGTCTCGCTCCTGGAAGATG |
| Mouse Metrnl | CTGGAGCAGGGAGGCTTATTT | GGACAACAAAGTCACTGGTACAG |
| Mouse IL1β | GCAACTGTTCCTGAACTCAACT | ATCTTTTGGGGTCCGTCAACT |
| Mouse IL6 | TAGTCCTTCCTACCCCAATTTCC | TTGGTCCTTAGCCACTCCTTC |
| Mouse MCP1 | GGCTCAGCCAGATGCAGTTAA | CCTACTCATTGGGATCATCTTGCT |

Suppl. Table 2. Antibodies used for Western blotting.

| Antigen/Target | Source | Stock | Dilution |
| --- | --- | --- | --- |
| Metrnl | R&D systems | MAB78672 | 1:1000 |
| p-MLC | Cell signaling | 3675 | 1:1000 |
| MLC | Cell signaling | 3672 | 1:1000 |
| MLCK | Abcam | ab76092 | 1:1000 |
| Flag | Abmart | M20008 | 1:1000 |
| p-IκBα | Beyotime | AF5851 | 1:1000 |
| IκBα | Beyotime | AF5204 | 1:1000 |
| p-p65 | Beyotime | AF5881 | 1:1000 |
| P65 | Beyotime | AF5243 | 1:1000 |
| p-IKKβ | Beyotime | AI139 | 1:1000 |
| IKKβ | Beyotime | AF7200 | 1:1000 |
| Occludin | Proteintech | 27260-1-AP | 1:1000 |
| ZO2 | Proteintech | 18900-1-AP | 1:1000 |
| Claudin 3 | Proteintech | 16456-1-AP | 1:1000 |
| Tubulin | Beyotime | AT819 | 1:2000 |
| Actinb | Beyotime | AA128 | 1:5000 |

Suppl. Table 3. General physiological characteristics of patients and control subjects.

| Parameters | Patients | Control subjects | P |
| --- | --- | --- | --- |
| Number of subjects | 15 | 21 | - |
| Age (years) | 53.67±12.79 | 50.67±13.34 | 0.50 |
| Gender (M/F) | 8/7 | 11/10 | - |
| WBC (109) | 8.76±26.11 | 5.29±3.37 | 0.55 |
| IL-6 (pg/ml) | 175.01±362.36 | 8.08±7.58 | 0.04 |
| CRP (ng/ml) circumference(cm) | 51.25±46.4 | 11.38±16.80 | 0.00 |

M/F, male/female; WBC, white blood cell; CRP, C-reactive protein.
