# Supplementary material for: Metrnl protects intestinal barrier function by regulating tight junctions via the IKKβ/IκBα/NFκB/MLCK/MLC signaling pathway
Source: Cell Death Discov. 2025 Apr 8;11:155. doi: 10.1038/s41420-025-02457-1 (PMC11979045; doi:10.1038/s41420-025-02457-1)
Supplement: Supplementary file 2 — Supporting material [file 41420_2025_2457_MOESM2_ESM.docx]

Supporting material

**Figure 1**

Metrnl





36Kd

25Kd

Marker

Marker

kidney

muscle

colon

brain

stomach

lung

heart

spleen

Tubulin


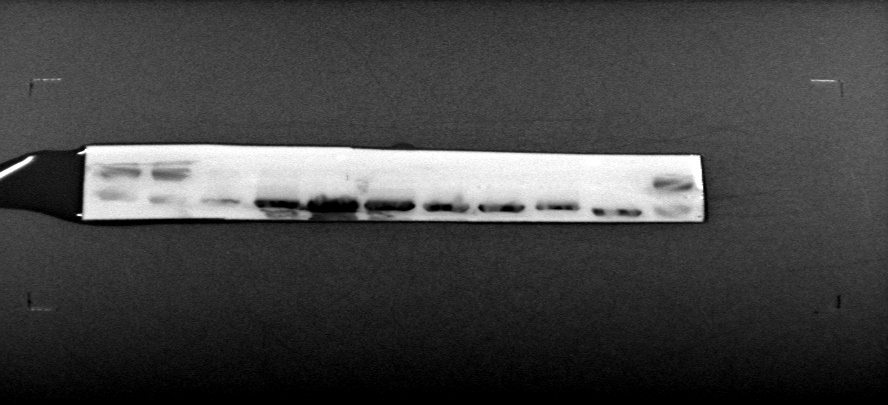


kidney

muscle

colon

stomach

lung

brain

spleen

heart

**72Kd**

**55Kd**

Marker

Marker

GAPDH


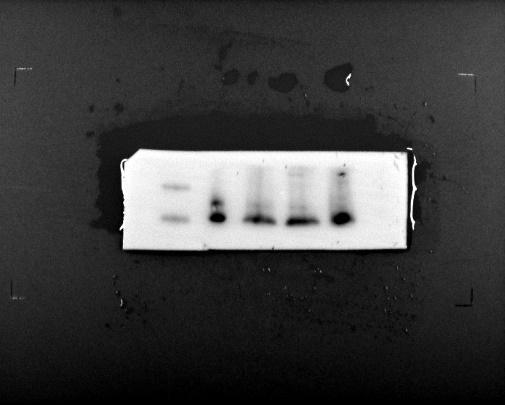


lleum

colon

**55Kd**

**36Kd**

Marker

duodenum

jejenum

Metrnl


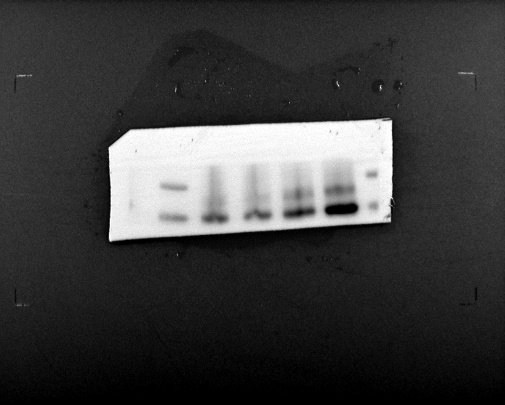


colon

lleum

Marker

**55Kd**

**36Kd**

duodenum

jejenum

**Figure 3**

**A**

Claudin 2





**25Kd**

Marker

——————————— ——————————

WT IE-Metrnl^-/-^

Claudin3





**25Kd**

Marker

Marker

——————————— ——————————

WT IE-Metrnl^-/-^

Tubulin（55 KDa） WT IE-Metrnl^-/-^





**55Kd**

——————————— ——————————

WT IE-Metrnl^-/-^

Marker

Zo2

——————————— ——————————

WT IE-Metrnl^-/-^





**170Kd**

**130Kd**

**E**

p-MLC


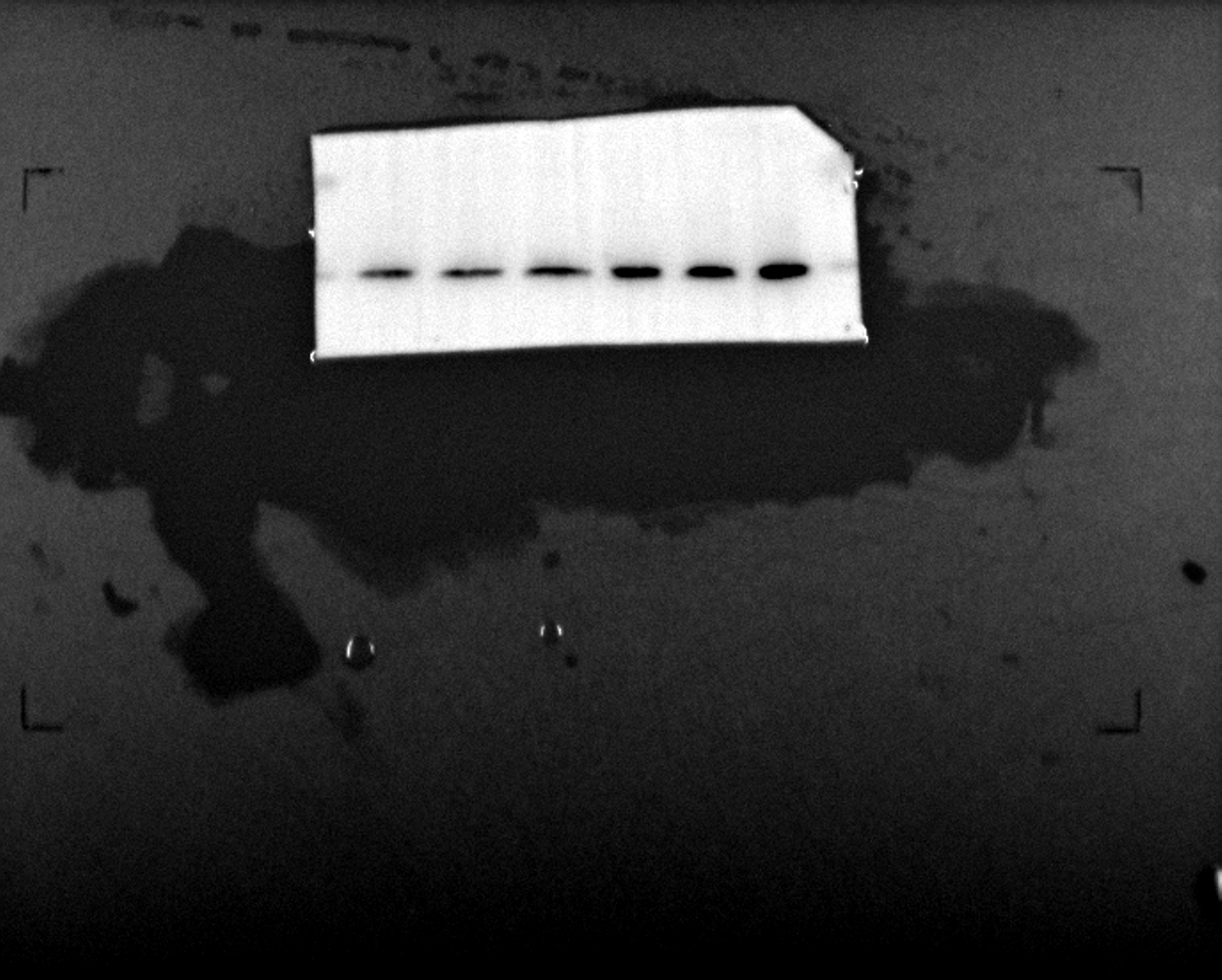


18Kd

——————————— ——————————

WT + endotoxin IE-Metrnl^-/-^ + endotoxin

MLC


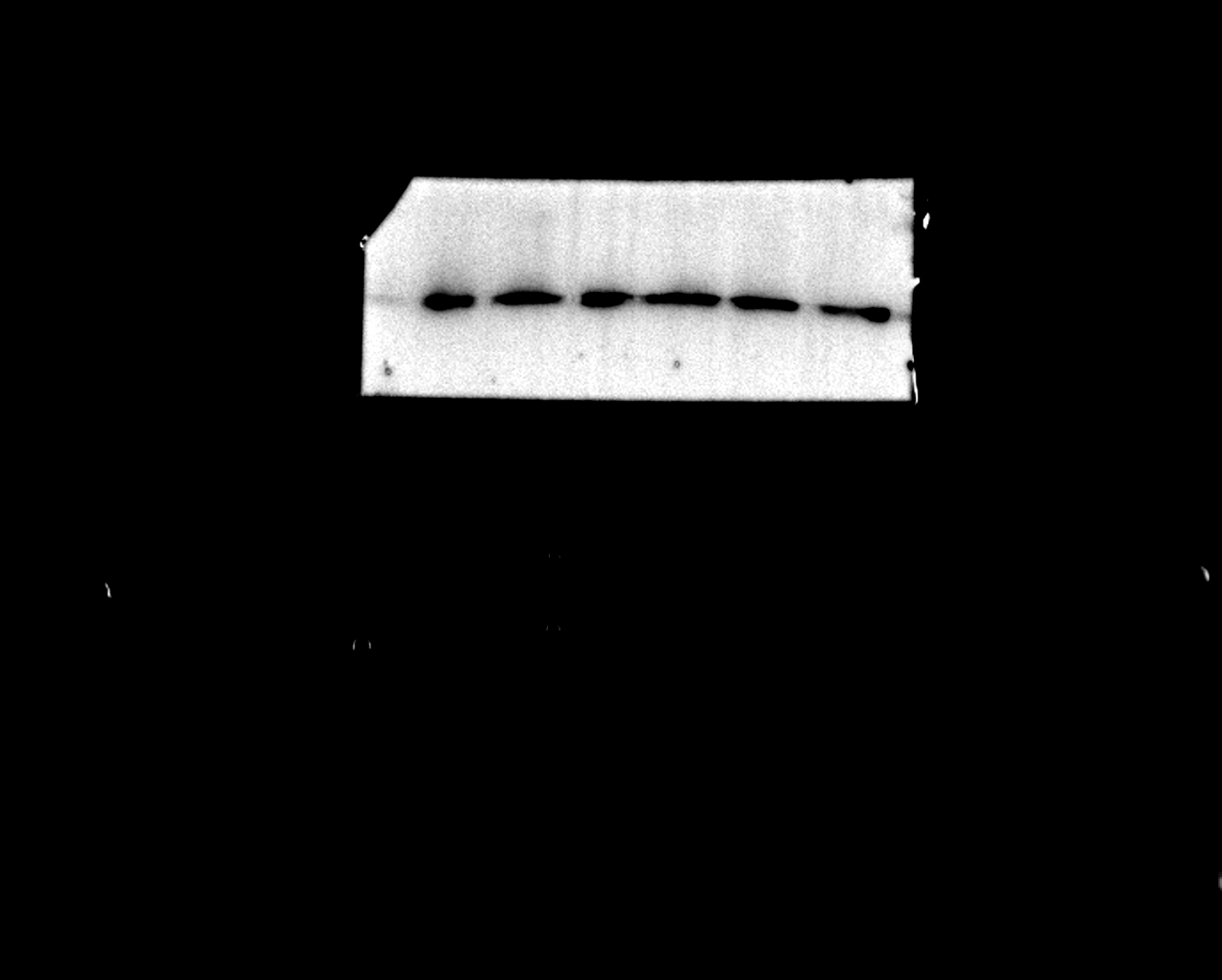


18Kd

——————————— ——————————

WT + endotoxin IE-Metrnl^-/-^ + endotoxin

Actinb


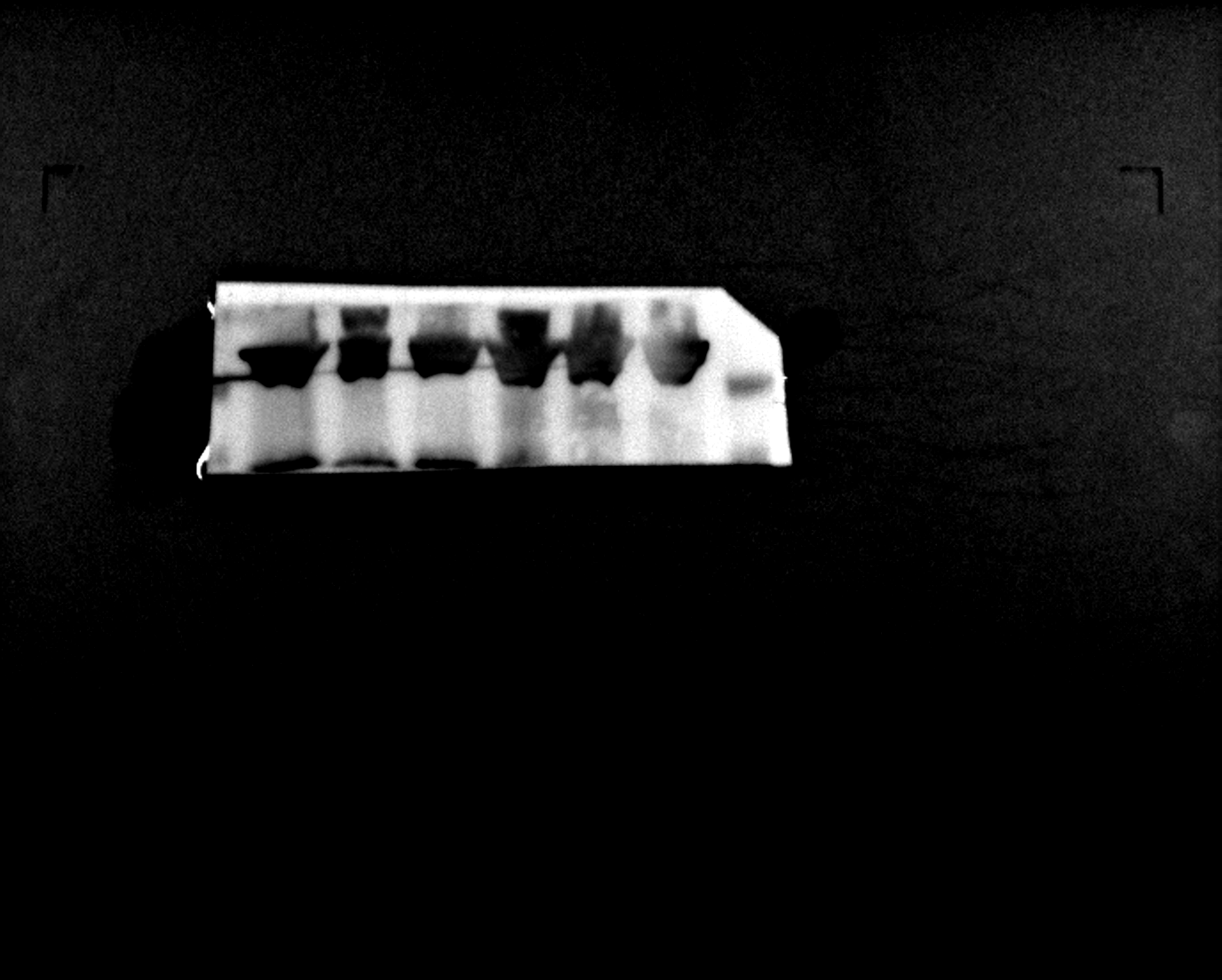


——————————— ——————————

WT + endotoxin IE-Metrnl^-/-^ + endotoxin

**F**

MLC


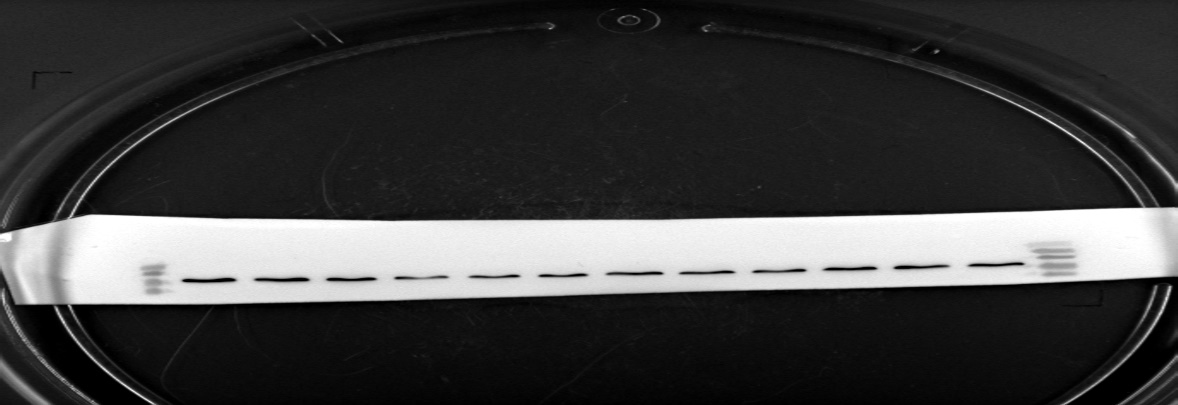


Marker

18Kd

——————— ——————— —————— ——————

Scr shRNA+PBS Metrnl shRNA Scr shRNA+ Metrnl shRNA+

+PBS endotoxin endotoxin

MLCK





——————— ——————— —————— ——————

Scr shRNA+PBS Metrnl shRNA Scr shRNA+ Metrnl shRNA+

+PBS endotoxin endotoxin

p-MLC

**
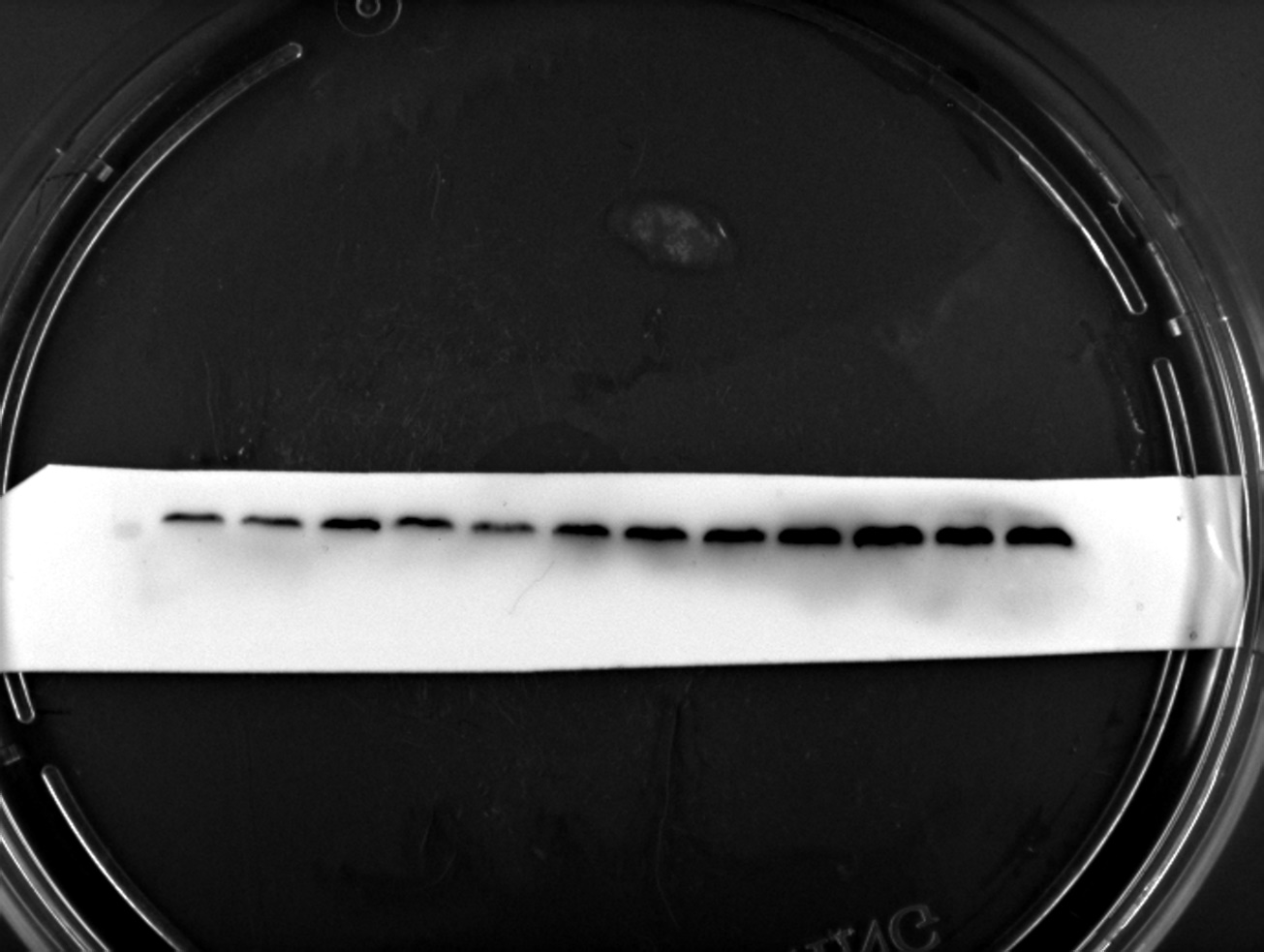
**

——————— ——————— —————— ——————

Scr shRNA+PBS Metrnl shRNA Scr shRNA+ Metrnl shRNA+

+PBS endotoxin endotoxin

**Figure 4**

**C**

Metrnl


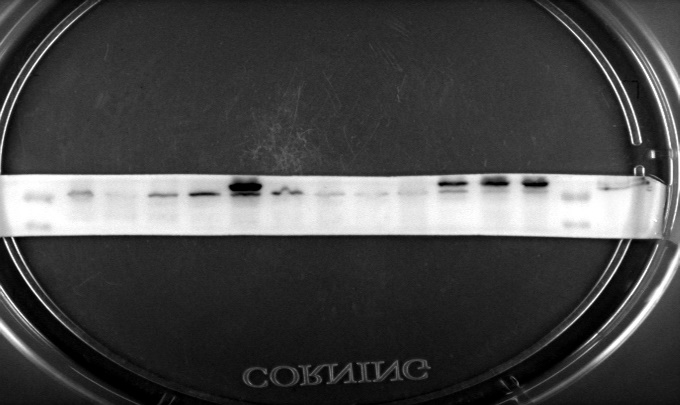


36Kd

25Kd

——— ———

WT IE-Metrnl OE

Flag


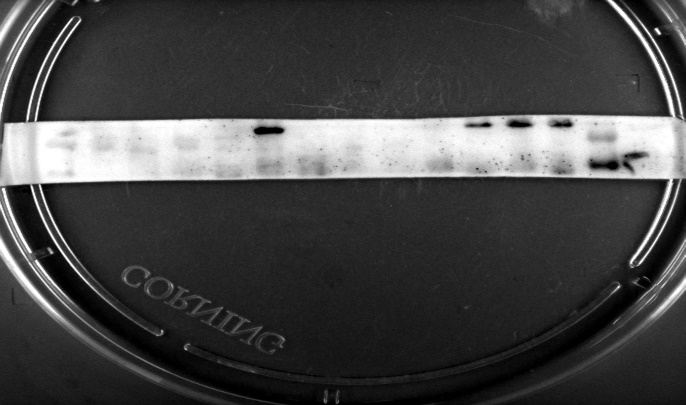


36Kd

25Kd

——— ———

WT IE-Metrnl OE

**Figure 7**

**A**

p-IkBα





55Kd

36Kd

——— ———

WT IE-Metrnl^-/-^

IkBα


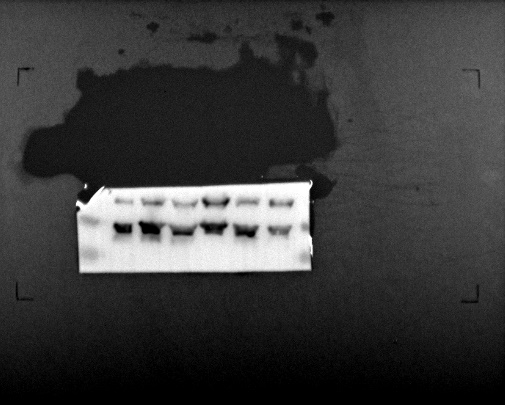


——— ———

WT IE-Metrnl^-/-^

55Kd

36Kd

p-p65





——— ———

WT IE-Metrnl^-/-^

72Kd

65Kd

p-IKKβ


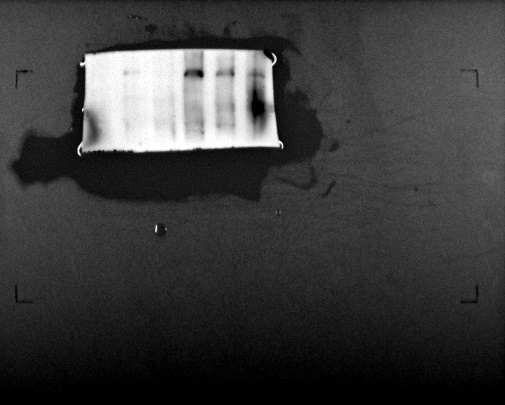


——— ———

WT IE-Metrnl^-/-^

80Kd

72Kd

IKKβ





——— ———

WT IE-Metrnl^-/-^

80Kd

72Kd
